# From disease to syndrome pattern: a study on the differentiation of traditional Chinese medicine syndrome patterns in asthma using multi-omics characterization

**DOI:** 10.3389/fimmu.2026.1922660

**Published:** 2026-09-15

**Authors:** Jing Zhang, Weilin Ying, Lian Liao, Xin Jing, Yuting Zhou, Li Yue, Boda Zhang

**Affiliations:** 1 Affiliated Hospital of North Sichuan Medical College, Nanchong, China; 2 Biotechnology Innovation Drug Application and Transformation Key Laboratory of Sichuan Province, North Sichuan Medical College, Nanchong, China; 3 Institute of Nanomedicine Innovation and Translational Research, Affiliated Hospital of North Sichuan Medical College, Nanchong, China; 4 Institute of Integrated Traditional Chinese and Western Medicine, North Sichuan Medical College, Nanchong, China

**Keywords:** bronchial asthma, Lung-Qi Deficiency (LQD) pattern, multi-omics, Phlegm-Dampness Obstructing the Lungs (PZLP) pattern, traditional Chinese medicine syndrome

## Abstract

**Background:**

Bronchial asthma is a highly heterogeneous chronic inflammatory airway disorder. Syndrome differentiation-guided Traditional Chinese Medicine (TCM) treatment for asthma delivers distinct advantages, including fewer adverse reactions and superior anti-inflammatory efficacy. Nevertheless, the intrinsic biological mechanisms underlying TCM asthma syndromes remain largely uncharacterized, restricting the objective and standardized differentiation of TCM subtypes.

**Methods:**

We integrated transcriptomic, proteomic, metabolomic and oral microbiome data from asthma patients and healthy controls to map asthma molecular and microbiome landscapes. Two representative TCM subtypes—Phlegm-Dampness Obstructing the Lungs (PZLP) and Lung-Qi Deficiency (LQD)—were stratified and compared via differential analysis, WGCNA, LEfSe and random forest machine learning to dissect shared asthma-related molecular features, subtype-specific molecular disparities and potential objective diagnostic biomarkers.

**Results:**

Comprehensive multi-omics and oral microbiome comparisons revealed correlational molecular discrepancies between asthma patients and healthy individuals. Our omics data suggest that asthma-related molecular alterations are tightly associated with three core pathological cascades: dysregulated glycerophospholipid metabolism, potential excessive activation of the NF-κB inflammatory signaling pathway, and impaired phagosome function. Beyond the universal disease-associated molecular signatures of asthma, prominent metabolic and inflammatory phenotypic divergences were detected between the two TCM subtypes. The PZLP subtype showed correlational omics signatures suggestive of elevated lipogenic activity, aberrant MAPK inflammatory pathway activation, and massive intracellular lipid accumulation. In contrast, the LQD subtype exhibited correlated downregulation of lipid transporter genes (e.g., ABCA13), which hypothetically implies compromised lipid transport capacity and disrupted cell membrane homeostasis. Oral microbiota profiling demonstrated profound structural remodeling in asthmatic patients. Although PZLP and LQD patients shared analogous overall microbial community structures, their microbial functional pathways diverged substantially: the LQD group was enriched in methane and glycerol metabolic pathways, whereas the PZLP group displayed overrepresentation of serotonergic and dopaminergic synaptic regulatory pathways. A diagnostic classification model built on differential microbial biomarkers achieved an area under the receiver operating characteristic curve (AUC) of 0.889, demonstrating robust discriminative capacity for distinguishing the two TCM syndromes.

**Conclusion:**

This study delineates correlational immunometabolic and oral microbiome signatures of asthma and uncovers potential subtype-specific molecular and microbial markers for two classic TCM asthma patterns. These observations provide testable mechanistic hypotheses for interpreting the molecular pathogenesis of asthma and could supply a preliminary theoretical foundation for objective TCM syndrome differentiation, personalized intervention and targeted asthma therapeutic development.

## 1 Introduction

Bronchial asthma ranks as the second leading cause of mortality among chronic respiratory diseases, impacting an estimated 300 million individuals globally and constituting a substantial public health burden (1). The pathophysiological process of bronchial asthma is closely related to airway remodeling, immune inflammation, neural regulation, protein kinases, and genetics (2). Genetic predispositions to immune dysregulation can amplify underlying chronic airway inflammation (3); external allergens and pathogens can damage the airway epithelial barrier and trigger the release of epithelial cytokines, leading to inflammation. Abnormal immune responses and repair processes then cause structural changes in the airways of all sizes (4). Currently, the treatment of bronchial asthma focuses primarily on symptom control and reducing acute exacerbations; however, long-term medication use may lead to drug resistance (5), and the recurrence rate is high after discontinuation. Prolonged use of glucocorticoids can affect growth and development, increasing the risk of conditions such as osteoporosis (6) and avascular necrosis of the femoral head (7), and has limited efficacy in reversing established airway remodeling (8).

The treatment of bronchial asthma using TCM syndrome differentiation offers advantages such as fewer side effects, superior anti-inflammatory and antispasmodic effects (9), improved immune function (10), and personalized treatment. TCM syndrome differentiation and treatment, an ancient precision medicine from China, classifies a single disease into various syndromes based on etiology and disease stage. Physicians use observation, auscultation, inquiry, and palpation to differentiate syndromes and create tailored treatments. In a multicenter, randomized, double-blind, placebo-controlled trial with 132 patients from three hospitals, Su Huang Cough Relief Capsules combined with ICS+LABA for 14 days improved cough relief, reduced blood eosinophil counts, IgE, and major basic protein levels, and enhanced small airway function. Animal studies confirmed its effectiveness in reducing airway inflammation, mucus secretion, and collagen deposition (11).

Unlike single-target Western therapeutic agents, TCM herbal formulations exert pharmacological effects via multi-component, multi-target, and multi-pathway regulatory networks (12). Meanwhile, the biological material basis of diverse TCM syndromes has not been fully elucidated. Consequently, clinical TCM syndrome diagnosis largely relies on physicians’ subjective experience and empirical judgment. At present, standardized qualitative and quantitative biological indicators are absent to differentiate various TCM syndromes and guide targeted herbal formulation selection.

High-throughput sequencing and systems biology have propelled multi-omics to become a key tool for investigating patterns in TCM. Multi-omics technologies can elucidate their biological mechanisms from multiple perspectives and aid in the screening of potential biomarkers (13). Among these, Wu Gaosong (14) et al. explored the mechanisms and biomarkers of the “Cold Congestion with Qi Stagnation” and “Qi Stagnation with Blood Stasis” patterns in coronary heart disease through a combined approach of proteomics, metabolomics, and network pharmacology. Yang Guang and colleagues used multi-omics to study the “Phlegm-Stasis and Blood-Stasis” pattern in coronary heart disease (15). Ji Shaoxiu’s team applied proteomics to identify biomarkers for the “Spleen-Kidney Yang Deficiency” pattern in HIV-induced immunological non-responders (HIV-INR) (16). Li Jiansheng’s group analyzed metabolomics and lipidomics to differentiate metabolites and pathways in COPD patterns. While multi-omics research has been conducted on various TCM disease patterns like COPD, there’s a lack of such studies on bronchial asthma patterns. LQD and PZLP are common asthma patterns, categorized as “deficiency” and “excess,” respectively, but their molecular mechanisms and microbiome foundations are unclear, with few comparative studies available (17).

Therefore, this study first establishes a comprehensive multi-omics profile of asthma and HC. Building on this foundation, it conducts an in-depth comparison of the differences in transcriptomic, proteomic, metabolomic, and oral microbiome profiles between PZLP and LQD, and constructs multidimensional networks to elucidate the molecular mechanisms underlying PZLP and LQD. The aim is to provide a scientific basis for the objectification and precision of TCM syndrome differentiation in asthma.

## 2 Manuscript formatting

### 2.1 Methodology: study design and participant selection

This cross-sectional case-control multi-omics study recruited a total of 49 participants from the Affiliated Hospital of North Sichuan Medical College between 1 July 2025 and 1 February 2026, including 13 healthy control subjects, 18 patients diagnosed with LQD-type asthma, and 18 patients diagnosed with PZLP-type asthma.

The Western medical diagnosis of asthma is based on the *Guidelines for the Prevention and Treatment of Bronchial Asthma (2024 Edition)* (18): ① Typical symptoms and signs: (1) Repeated instances of wheezing, breathlessness, chest constriction, and coughing, happening more often during nighttime and early morning, with varying intensity over time, and frequently linked to contact with allergens, cold air, physical or chemical irritants, upper respiratory infections, or physical activity; (2) In episodes and certain cases of uncontrolled chronic persistent asthma, wheezing can be heard throughout both lungs, with an extended expiratory phase; these symptoms may improve with treatment or on their own. ② Evidence of airflow restriction that can be reversed: (1) Positive bronchodilator test; (2) Positive bronchial provocation test. Bronchial asthma can be diagnosed when the aforementioned symptoms and signs are observed, in addition to at least one objective sign of limited airflow, and after ruling out wheezing, shortness of breath, chest tightness, and cough due to other conditions.

TCM diagnostic criteria for asthma: In accordance with the *Diagnostic Criteria for Traditional Chinese Medicine Syndromes of Bronchial Asthma (2016 Edition)* (19), Two attending physicians or higher-ranked TCM practitioners independently performed syndrome differentiation for all participants. Consistent diagnostic results were directly adopted; inconsistent judgments were arbitrated by one associate chief physician or senior TCM specialist, whose conclusion served as the final diagnostic standard. Although Kappa consistency testing was not implemented for inter-rater reliability evaluation, all diagnostic discrepancies were resolved via third-party expert arbitration. All enrolled asthmatic patients were in clinical remission or stable phase without acute exacerbations within 4 weeks prior to recruitment. No patients received oral or intravenous glucocorticoids within 2 weeks before sample collection. Patients with concurrent dual LQD and PZLP manifestations or complicated additional TCM syndromes (e.g., blood stasis, yin deficiency) were excluded; only patients with a single pure LQD or PZLP syndrome were eligible for enrollment.

Lung Qi Deficiency Syndrome:

Major Symptoms: Shortness of breath, cough, mental fatigue, weakness, spontaneous sweating, pale tongue, and a fine pulse.
Minor Symptoms: Wheezing, chest tightness, susceptibility to colds, pale complexion, and difficulty expectorating phlegm.
Tongue and Pulse: Swollen tongue, tooth marks on the tongue, white tongue coating; weak or deep pulse.

A definitive diagnosis can be established when the major symptoms are present along with at least two minor symptoms, combined with tongue and pulse observations.

Syndrome of Phlegm-Dampness Obstructing the Lungs:

Major Symptoms: Shortness of breath, wheezing, chest tightness, difficulty breathing, coughing, copious sputum, white sputum, thin and clear sputum, white tongue coating.
Minor Symptoms: Difficulty expectorating phlegm, wheezing in the throat, phlegm that is easily coughed up, poor appetite, loss of appetite, fatigue, epigastric fullness, loose stools.
Tongue and Pulse: Swollen tongue, tooth marks on the tongue, pale-red tongue body, greasy tongue coating, slippery pulse, and taut pulse.

The major symptoms need to be evident, along with at least two minor symptoms; together with tongue and pulse observations, a diagnosis can be established.

Inclusion Criteria

1. Patients meeting the Western medical diagnostic criteria for bronchial asthma;
2. Patients meeting the aforementioned TCM diagnostic criteria, with a syndrome differentiation of Lung Qi Deficiency or Phlegm-Dampness Obstructing the Lungs;
3. Complete clinical records;
4. No gender restrictions; age 18–60 years (including 18 and 60);
5. Individuals who willingly choose to join the study and have provided their informed consent.

Exclusion Criteria

1. Individuals diagnosed with simultaneous malignant tumors;
2. Patients undergoing invasive ventilation therapy;
3. Patients with other respiratory diseases, such as pulmonary tuberculosis;
4. Patients with severe, uncontrolled organic lesions or infections, such as decompensated cardiac, pulmonary, or renal failure;
5. Patients with a severe bleeding tendency or hemorrhagic disorders;
6. Patients lacking legal capacity due to mental illness or intellectual disability;
7. Patients who are pregnant, breastfeeding, may be pregnant, or planning to become pregnant;
8. Patients who have participated in or are currently participating in other clinical trials within the past 3 months, or those deemed ineligible by the investigator for any other reason.

The healthy control group is made up of patients without recent respiratory symptoms, infections, or chronic respiratory conditions. The schematic overview of this study is presented in **Figure 1**.

**Figure 1:**
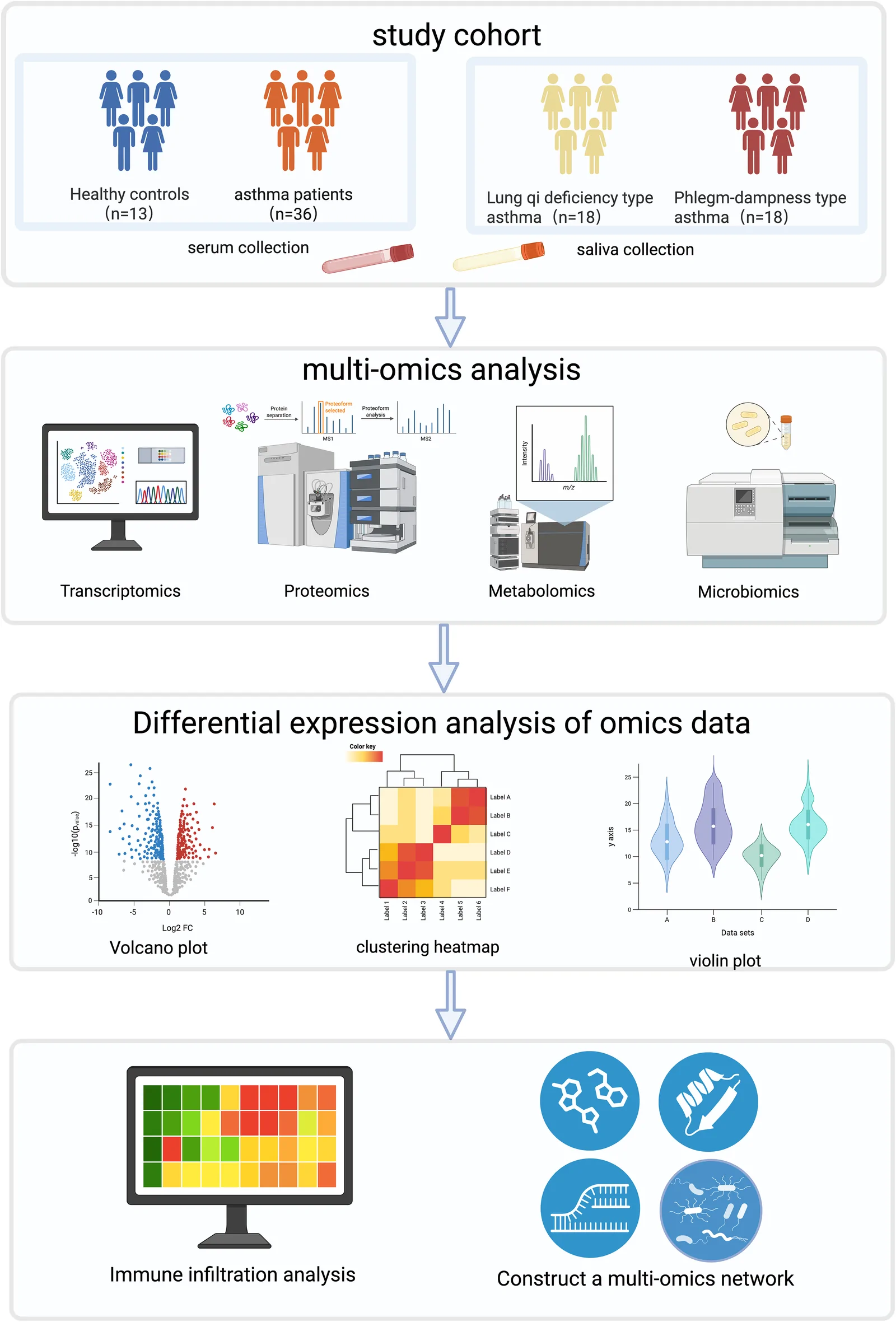
Study design flowchart. Figure created with BioRender.com.

Every participant is required to take pulmonary function tests and have a complete blood count. They must also sign an informed consent form after comprehending all pertinent study information. The Ethics Committee of the Affiliated Hospital of North Sichuan Medical College has additionally given formal approval to this study (Approval No.: 2025ER497-1).

#### 2.1.1 Sample collection

In the morning, fasting venous blood was drawn from all participants using serum separation tubes containing coagulant additives. Following 60 minutes of clotting at room temperature, the samples were spun at 3000 rpm and 4 °C for 10 minutes. Then, 200 μL of the supernatant serum was divided into pre-labeled 2 mL centrifuge tubes. Samples were quickly frozen in liquid nitrogen for 5–10 minutes and stored long-term at −80 °C.

Oral Microbiome: Participants rinsed their oral cavities with purified water; saliva was accumulated at the floor of the mouth after a 30-min waiting period, and 3–5 mL saliva was collected via repeated expectoration at 60-second intervals. One milliliter of saliva was transferred to a 5 mL centrifuge tube, rapidly frozen using liquid nitrogen for 5 to 10 minutes, and kept at −80 °C.

#### 2.1.2 Transcriptomic profiling

Total RNA was isolated using the Trizol method, which employs phenol-guanidine isothiocyanate, and then it was dissolved in DEPC-treated ultrapure water. The concentration of RNA was measured with a Qubit fluorometer, and the integrity of RNA (RQN value) was evaluated using a Qsep400 high-throughput biochip analyzer to ensure the quality standards for library construction.

Oligo(dT) magnetic beads were applied to enrich poly(A)-tailed mRNA. Fragmentation buffer was used to fragment purified mRNA, and random hexamer primers were employed to reverse-transcribe the first-strand cDNA. The following steps involved end repair, adding dA tails, and attaching sequencing adapters. The ligation products were then purified using DNA magnetic beads, and fragments with insert sizes between 250 and 350 bp were selected. After PCR amplification, libraries underwent secondary purification. Following quality control, libraries were pooled according to their effective concentration and intended sequencing throughput, and paired-end 150 bp (PE150) sequencing was conducted using the Illumina NovaSeq X Plus platform.

The fastp software was used to preprocess raw sequencing reads: Sequences containing sequencing adapters, more than 10% N bases, and over 50% low-quality bases (Q ≤ 20) were eliminated to generate high-quality clean reads. HISAT2 was used to map clean reads against the human reference genome, and featureCounts was used to quantify gene expression, generating a matrix of raw read counts. These raw counts were directly used as input for DESeq2 with default parameters to perform differential expression analysis, and P-values were adjusted using the Benjamini-Hochberg method. FPKM values were calculated from the raw counts for visualization purposes only and were not involved in the differential expression analysis. Differential gene screening thresholds were set as |log_2_(fold change)| > 1 and adjusted *P* < 0.05. Hypergeometric testing was adopted for Gene Ontology (GO) and Kyoto Encyclopedia of Genes and Genomes (KEGG) enrichment analyses. rMATS analyzed five categories of alternative splicing events (SE, RI, MXE, A5SS, A3SS); GATK performed single-nucleotide polymorphism (SNP) detection, and STAR-Fusion identified fusion genes. Differentially expressed genes had their protein-protein interaction networks built using the STRING database. ClusterProfiler was used to perform gene set enrichment analysis (GSEA), while the WGCNA R package was utilized for weighted gene co-expression network analysis (WGCNA).

#### 2.1.3 Proteomic profiling

A 50 μL serum sample was processed with magnetic bead enrichment for low-abundance proteins. A total of 20 μL of magnetic beads were pre-washed with equilibration buffer, combined with serum diluted in the same buffer, and incubated at 37 °C with shaking at 1000 rpm for 1 hour in a thermomixer. The tubes were set on a magnetic separator for at least 2 minutes to fully eliminate any remaining serum supernatant without drawing up the magnetic beads. A volume of 200 μL washing buffer was added, followed by 5 min of room-temperature mixing and repeated magnetic separation for at least 2 min to discard liquid; this washing cycle was repeated three times. To achieve complete protein lysis, 50 μL of lysis buffer was introduced, and the samples were heated at 95 °C with shaking at 1000 rpm for 10 minutes, then allowed to cool to ambient temperature. A 10 μL mixture of trypsin and Lys-C working solution was added for enzymatic digestion at 37 °C, 1000 rpm for 3 h. Digestion was terminated by adding 5 μL stop solution followed by vortex mixing. After magnetic separation for ≥2 min, the digested peptide supernatant was transferred to a new centrifuge tube. Peptides were desalted with a C18 tip column, vacuum freeze-dried, and reconstituted in 0.1% formic acid aqueous solution. Peptide concentration was determined via the BeyoBCA colorimetric peptide quantification kit, and all samples were normalized to a final concentration of 100 ng/μL using 0.1% formic acid solution.

Chromatographic separation was performed on a Vanquish Neo UHPLC system equipped with a dual trap-elution column setup: trap column PepMap Neo Trap Cartridge (300 μm × 5 mm, 5 μm); analytical column Easy-Spray™ PepMap™ Neo UHPLC column (150 μm × 15 cm, 2 μm). Column temperature was maintained at 55 °C, injection volume 200 ng, flow rate 2.5 μL/min, linear gradient duration 13 min, total run time 14 min. Mass spectrometric detection was conducted on an Orbitrap Astral high-resolution mass spectrometer under positive ion mode with data-independent acquisition (DIA). MS full scan range covered m/z 380–980 at a resolution of 240,000 (m/z = 200), normalized AGC target 500%, maximum ion injection time 5 ms. DIA MS/MS acquisition contained 299 scanning windows with an isolation width of 2 Th, HCD collision energy 25%, normalized AGC target 500%, maximum ion injection time 3 ms.

Raw MS data were processed using the library-free DIA-NN v1.8.1 algorithm. The human UniProt reference proteome database (uniprotkb_proteome_UP000005640_human_83385_20250224.fasta, 83,385 total sequences) was applied to build spectral libraries via deep neural network modeling, with the Match Between Runs (MBR) function activated. False discovery rate (FDR) was controlled below 1% at both protein and precursor ion levels; only qualified identification results were subjected to subsequent quantitative analysis.

#### 2.1.4 Non-targeted metabolomic profiling

Each 50 μL serum aliquot was thawed on ice and vortex-mixed for 10 s. A total of 300 μL internal standard-containing extraction solvent (acetonitrile:methanol = 1:4, v/v) was added, followed by 3 min continuous vortexing. The mixture was centrifuged at 12,000 rpm and 4 °C for 10 min. A volume of 200 μL supernatant was transferred to a new tube and incubated at −20 °C for 30 min. After secondary centrifugation under identical conditions for 3 min, 180 μL supernatant was reserved for LC-MS/MS detection.

Metabolite separation was achieved on a Waters ACQUITY Premier HSS T3 analytical column (1.8 μm particle size, 2.1 mm × 100 mm). Ultrapure water with 0.1% formic acid constituted mobile phase A, and mobile phase B was acetonitrile with 0.1% formic acid added. Linear gradient elution schedule: 0–2 min, 5%–20% B; 2–5 min, 20%–60% B; 5–6 min, 60%–99% B; 6–7.5 min, constant 99% B; 7.5–7.6 min, 99%–5% B; 7.6–10 min, constant 5% B. A column temperature of 40 °C was set, with a flow rate of 0.4 mL/min and an injection volume of 3 μL. Both ESI+ and ESI− modes utilized the identical gradient program.

Parameters for the ESI ion source include a spray voltage of 3.8 kV for ESI+ and 3.4 kV for ESI−, with a sheath gas flow of 60 Arb and an auxiliary gas flow of 20 Arb. The ion transfer tube is set to 320 °C, and the vaporizer is at 350 °C. Full-scan MS spectra were acquired across m/z 70–1000 with a resolution of 60,000. Data-dependent MS/MS fragmentation (TopN = 10) was performed with stepped collision energies of 30 V, 40 V, and 50 V.

ProteoWizard was used to convert raw LC-MS data into mzML format, and XCMS software handled peak picking, alignment, and retention time correction. Features with over 50% missing values across all samples were eliminated; missing values below 50% were filled via KNN interpolation, while features with ≥50% missing data were imputed with one-fifth of the minimum peak intensity of the dataset. Support vector regression (SVR) was used to correct signal intensity. Metabolite annotation was completed by matching against an in-house standard library and public databases (HMDB, KEGG, Metlin), with identification thresholds composite score ≥0.5 and QC sample coefficient of variation (CV) < 0.3. The prcomp function in R was used to perform unsupervised principal component analysis (PCA), with raw data being log_2_-transformed and mean-centered before analysis. Orthogonal partial least squares discriminant analysis (OPLS-DA) was performed using the MetaboAnalystR package with log_2_ transformation and UV normalization; 200-fold cross-validation was adopted to avoid model overfitting. Pairwise differential metabolite screening criteria: variable importance in projection (VIP) > 1 and *P* < 0.05 (two-tailed t-test); one-way ANOVA was used for multi-group comparisons. Annotated metabolites were mapped to the KEGG Compound database for subsequent KEGG pathway enrichment analysis.

#### 2.1.5 Oral 16S rRNA microbiome sequencing

The CretMag™ Power Soil DNA Kit was used to extract total microbial genomic DNA from saliva samples. The integrity of the DNA was checked using 1% agarose gel electrophoresis, and its concentration and purity were measured with a NanoDrop 2000 spectrophotometer. Using primer pairs 515F/806R, the V4 hypervariable region of the 16S rRNA gene was amplified, with each sample having three technical replicates. PCR products from identical samples were pooled, separated on 2% agarose gels, and purified with the AxyPrep DNA Gel Extraction Kit. Sequencing libraries were constructed using the VAHTS^®^ Universal Plus DNA Library Prep Kit for MGI V2. After quality inspection, libraries were sequenced on the MGI paired-end sequencing platform. Raw sequencing reads were demultiplexed according to barcode and primer sequences. Fastp software filtered low-quality reads by removing adapter sequences, reads containing ambiguous N bases, sequences with over 40% low-quality bases (Q < 15) within a 4-base sliding window, reads with average quality < 20, polyG tails, and fragments shorter than 150 bp. High-quality paired-end reads were assembled via FLASH, and chimeric sequences were eliminated using vsearch against reference databases to generate valid amplicon tags. Deblur noise reduction integrated within QIIME 2 was applied to resolve amplicon sequence variants (ASVs). Using Mothur, taxonomic classification was conducted by aligning sequences with the SILVA database, with a similarity threshold of 0.8–1, resulting in community composition profiles at various taxonomic levels including phylum, class, order, family, genus, and species. Multiple sequence alignment was completed with MAFFT, and all samples were normalized to the minimum sequencing depth for subsequent comparative analysis.

R packages phyloseq and vegan were utilized to calculate alpha diversity indices (Observed_ASV, Chao1, ACE, Shannon, Simpson, Goods_coverage, PD_whole_tree), generate rarefaction and rank-abundance curves, and conduct intergroup difference testing via independent t-test or Kruskal-Wallis test. Beta diversity was evaluated through PCA, PCoA, and NMDS based on weighted/unweighted UniFrac distances; intergroup compositional disparities were validated via Anosim, MRPP, Adonis, and AMOVA algorithms. LEfSe pinpointed taxonomic biomarkers that showed significant differences in abundance between groups, with an LDA score greater than 4. Correlation analysis of the top 100 most abundant genera was performed using the FastSpar algorithm. Microbial functional prediction was conducted with PICRUSt2 based on the KEGG database; FAPROTAX annotated ecological functional profiles, and BugBase predicted microbial phenotypic traits (pathogenic potential, oxygen tolerance, etc.).Random forest classification models were constructed using the R package varSelRF to screen signature microbial taxa. The model adopted bootstrap aggregating resampling strategy: samples were drawn with replacement to build individual decision trees, and approximately 36.8% of original samples that were not involved in tree training were defined as out-of-bag (OOB) data. The OOB datasets, completely excluded from model training and feature selection, were used to estimate model classification performance. For random forest modelling, the analytical prerequisite that the sample size of each group should be no less than 15 was satisfied. Random forest classification models were constructed using the R package varSelRF to screen signature microbial taxa; bootstrap aggregation optimized model performance, with out-of-bag error estimating classification efficiency and ROC curves plotted for quantitative evaluation.

#### 2.1.6 The methodology for multi-omics integration

All transcriptomic, proteomic and metabolomic datasets underwent independent normalization prior to cross-omics integrated analysis. Pairwise two-omics combined analyses, including metabolome-proteome, metabolome-transcriptome and proteome-transcriptome association analyses, as well as comprehensive three-omics conjoint analysis were performed in this study. Transcripts and corresponding proteins were matched based on KEGG Orthology annotations retrieved from the SILVA database, and only molecular pairs with consistent quantitative signals across all samples were retained for subsequent integrated calculation. All genes and proteins were annotated to KEGG Orthology entries, while metabolites were assigned corresponding KEGG Compound IDs. Genes, proteins and metabolites co-localized within identical KEGG pathway topological maps were treated as matched molecular units for cross-omics correlation and joint-pathway analysis. Differential genes, differential proteins and differential metabolites were separately subjected to functional enrichment using hypergeometric tests. Pathways exhibiting enrichment signals in at least two omics layers were defined as shared KEGG pathways. The enrichment factor and raw P-value for each omics dataset were calculated, and shared pathways were visualized via bar graphs, bubble plots and full-scale pathway topology maps generated from KEGG KGML markup files. Pearson correlation coefficients between normalized quantitative values of multi-omics molecules were calculated using the built-in cor function within R. Only molecular pairs meeting the thresholds of absolute correlation coefficient > 0.8 and *P* < 0.05 were retained for downstream analysis to filter weak and non-significant associations. Nine-quadrant plots, density scatter diagrams and clustering heatmaps were generated to distinguish consistent or opposing expression trends of paired molecules across omics layers. Canonical correlation analysis and two-way orthogonal partial least-squares analysis were further implemented with the R packages CCA and OmicsPLS to explore global cross-omics associations. Two types of multi-omics interaction networks were constructed in the present work. KGML-derived pathway topological networks were visualized using information downloaded from the KEGG database, in which pathways, genes-proteins and metabolites were represented by distinct graphic shapes; up-regulated molecules were labelled red and down-regulated molecules were labelled blue. Significant correlated molecular pairs obtained from Pearson correlation screening were imported into Cytoscape to build undirected correlation networks. Node degree centrality was computed for all network nodes, molecules with high node-degree centrality within networks were identified as hub genes, hub proteins or hub metabolites exerting core regulatory roles in multi-omics crosstalk. Raw P-values from hypergeometric tests were preserved for GO and KEGG functional enrichment and visualized after -log10 transformation to illustrate enrichment magnitude. Rigorous correlation cut-offs were applied to mitigate false-positive results originating from massive pairwise comparisons among high-dimensional omics features. FDR-based multiple-testing correction was applied for all inter-group statistical analyses, including PCA-assisted grouping validation, Kruskal-Wallis test, Anosim, MRPP, AMOVA and PTR-based translation-efficiency analysis. All multi-omics integration computations were performed in R v4.2.0 utilizing the packages stats, psych, pheatmap, igraph, CCA, OmicsPLS and ggplot2 for statistical computation and graphical visualization.

#### 2.1.7 Statistical analysis of clinical data

Using SPSS 26.0 software, all clinical data underwent statistical analysis. Normally distributed continuous variables were presented as mean ± standard deviation (SD), whereas non-normally distributed data were shown as median with interquartile range (IQR). The Shapiro-Wilk test assessed normality of variable distributions. Between-group comparisons of normally distributed continuous variables adopted independent samples t-test, while non-normal data were analyzed via Wilcoxon rank-sum test. Statistical significance was determined by a two-tailed P-value of less than 0.05.

### 2.2 Results

#### 2.2.1 Characteristics of all participants

**Table 1** summarizes the baseline characteristics and pulmonary function parameters for the Asthma group (n = 36) and the Healthy Control (HC) group (n = 13). The two groups showed no statistically significant differences in age or BMI (*P* > 0.05), suggesting that the initial data were balanced and comparable. However, compared with the HC group, asthma patients had significantly elevated serum total IgE levels (*P* < 0.001). Blood cell analysis revealed significantly elevated absolute counts of eosinophils (EOS) and red blood cells (RBC) in the asthma group, suggesting the presence of systemic inflammation and hematopoietic-related changes in asthma patients. Pulmonary function tests revealed significant differences between groups: compared with asthma patients, the HC group performed better in terms of FEV1/FVC ratio, FEV1, and FVC levels (all *P* < 0.001), suggesting that asthma patients have clear airway obstruction and impaired pulmonary ventilation. Further comparison was conducted between the two asthma subgroups, PZLP (n = 18) and LQD (n = 18), with results shown in **Table 2**. Compared with the LQD group, the PZLP group had significantly higher BMI levels and EOS counts (*P* < 0.001). However, there were no significant differences in pulmonary function parameters—including the FEV1/FVC ratio, FEV1, and FVC levels—between the two asthma subtypes (all *P* > 0.05), suggesting that the degree of impaired pulmonary ventilation was not markedly different between the two groups.

**Table 1:** Demographic Information of Patients with HC and Asthma.

Variables	HC	Asthma	P
N	13	36	
Age/year	33.23 ± 5.10	37.58 ± 9.64	0.129
BMI	21.73 ± 1.62	21.67 ± 1.60	0.904
Blood Cell Detections			
WBC	6.73 ± 1.40	6.62 ± 1.45	0.806
EOS	0.10 ± 0.08	0.28 ± 0.11	0.001
NE	4.17 ± 1.01	4.139 ± 1.201	0.922
RBC	4.37 ± 0.316	4.86 ± 0.41	0.0004
PLT	275.3 ± 80.45	244.5 ± 57.29	0.143
Lung Function			
FEV1/FVC	0.80 ± 0.02	0.61 ± 0.059	<0.001
FEV1	3.44 ± 0.24	1.94 ± 0.45	<0.001
FVC	4.30 ± 0.31	3.16 ± 0.65	<0.001

**Table 2:** Baseline characteristics of asthma patients with LQD and PZLP patterns.

Variables	LQD	PZLP	P
N	18	18	
Age/year	38.56 ± 10.37	36.61 ± 9.05	0.553
BMI	20.5 ± 1.23	22.84 ± 0.95	<0.001
Blood Cell Detections			
WBC	6.449 ± 1.27	6.801 ± 1.63	0.475
EOS	0.24 ± 0.11	0.32 ± 0.11	<0.001
NE	3.974 ± 1.102	4.303 ± 1.303	0.420
RBC	4.788 ± 0.367	4.944 ± 0.456	0.264
PLT	254.9 ± 53.46	234.1 ± 60.56	0.281
Lung Function			
FEV1/FVC	0.632 ± 0.068	0.597 ± 0.044	0.082
FEV1	1.939 ± 0.479	1.959 ± 0.446	0.894
FVC	3.063 ± 0.6733	3.265 ± 0.638	0.319

#### 2.2.2 Identification of differentially expressed genes in bronchial asthma and healthy controls via transcriptomics (**Figures 2A–F**)

The PCA analysis showed that the first two principal components, PC1 at 13.59% and PC2 at 7.47%, together accounted for 21.06% of the overall variance. The HC and Asthma groups exhibited a clear separation in the two-dimensional space; although there was some overlap in samples, this suggests that the two groups possess significantly different overall transcriptomic profiles. Differential expression analysis identified a total of 355 DEGs, of which 256 were upregulated and 99 were downregulated in the Asthma group compared to the HC group, suggesting that asthma is characterized by expression patterns centered on immune activation. The radar plot of core differentially expressed genes showed that FIGN, TMSB4Y, PRY, and ZFY-AS1 were significantly upregulated in the Asthma group, while AGR2 and DKK1 were downregulated. According to GO enrichment analysis, DEGs were significantly associated with biological processes such as bacterial defense response, leukocyte migration, humoral antibacterial immunity, and activation of myeloid leukocytes; at the cellular component level, they were primarily enriched in cytoplasmic vesicles, secretory granules, and specific granules; at the molecular function level, they were enriched in sulfur compound binding, heparin binding, and peroxidase activity. KEGG pathway enrichment analysis further validated that DEGs were notably enriched in pathways related to environmental information processing, including cytokine-cytokine receptor interactions, the PI3K-Akt signaling pathway, and neuroactive ligand-receptor interactions; they were also enriched in human disease pathways such as fluid shear stress and atherosclerosis, as well as rheumatoid arthritis. WGCNA analysis revealed that the yellow and green-yellow modules were significantly positively correlated with the HC group, whereas the midnight-blue, blue, and turquoise modules were associated with the asthma state, suggesting a specific co-expression network reorganization in asthma. In summary, this study systematically identifies significant transcriptomic differences between the HC and Asthma groups, with core differences centered on immune inflammatory dysregulation, cell signaling and extracellular-matrix-related molecular signatures. These correlational findings lay a basis to generate testable hypotheses for asthma molecular pathophysiology.

**Figure 2:**
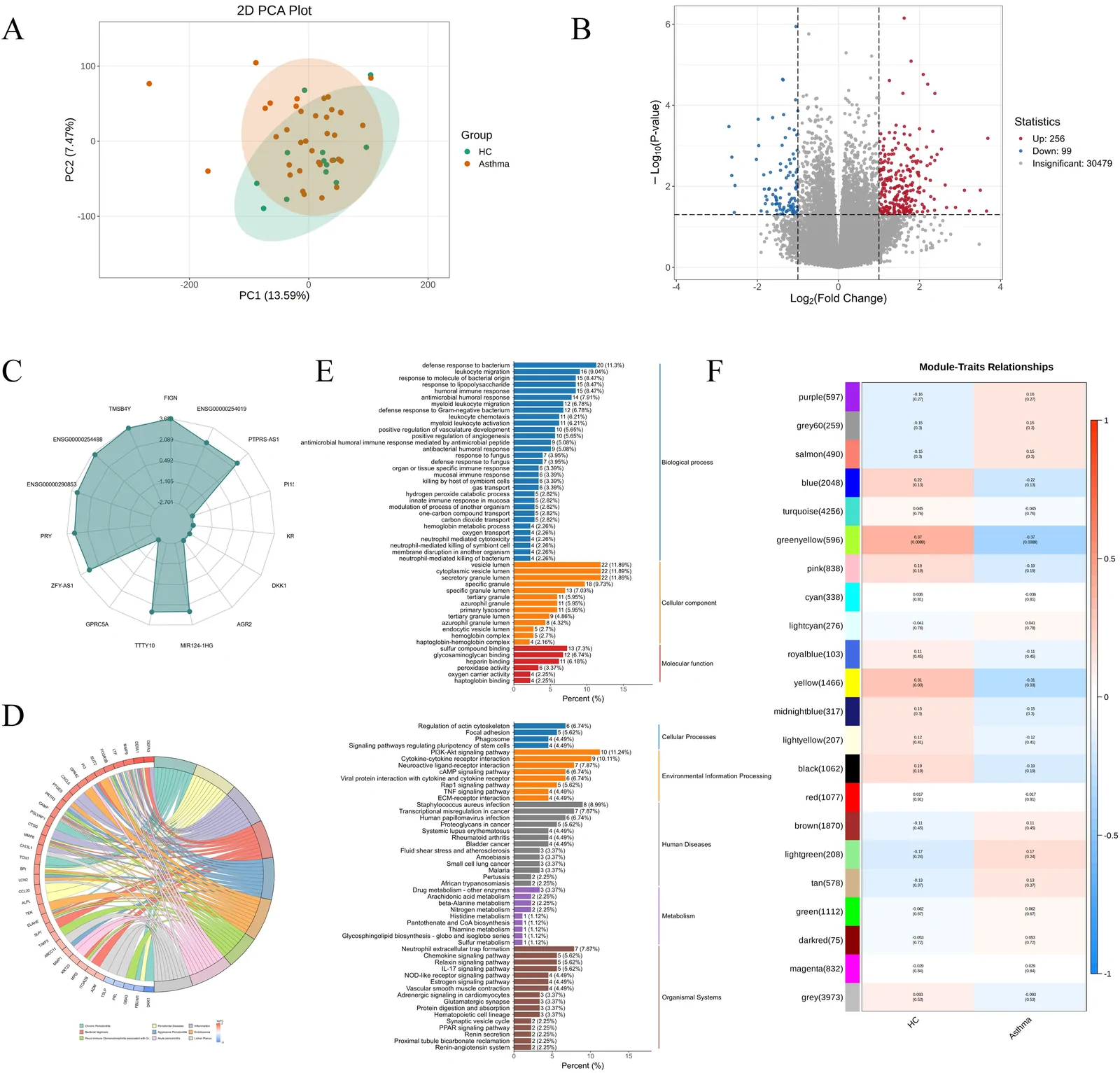
Transcriptomic Characteristics of Asthma Patients and Identification of Key Molecules and Pathways. **(A)** PCA plot of transcriptomic data from the asthma group and healthy control group. **(B)** Volcano plot showing differentially expressed genes: red represents upregulated genes, blue represents downregulated genes, and gray represents genes without significant changes. **(C)** Radar plot of core differentially expressed gene characteristics in asthma patients. **(D)** Circle plot of phenotypic association analysis for asthma. **(E)** GO functional enrichment analysis and KEGG pathway enrichment analysis of differentially expressed genes. **(F)** Heatmap showing the correlation between gene co-expression modules and disease phenotypes. Rows represent different WGCNA modules, columns represent the HC and Asthma groups, and colors indicate correlation coefficients (red for positive correlation, blue for negative correlation), illustrating the strength of association between each module and the disease state.

#### 2.2.3 Identification of differentially expressed proteins in proteomics data from bronchial asthma and healthy control groups (**Figures 3A–E**)

Using a screening threshold of |log_2_(Fold Change)| > 1 and *P* < 0.05, this study identified a total of 144 significant DEPs, of which 98 were upregulated and 46 were downregulated in the asthma group compared to the healthy control group. Principal component analysis revealed distinguishable expression patterns between the two groups, and the clustering heatmap showed clear separation of the two groups, indicating that the differentially expressed proteins can effectively distinguish between the two pathological states. The analysis of GO functional enrichment indicated that DEPs were notably enriched in essential biological processes, including the immune response, oxygen binding, and hemoglobin complexes, suggesting that dysregulated immune responses and abnormal oxygen metabolism are key pathological features of asthma; KEGG pathway analysis further revealed that differentially expressed molecules were primarily enriched in pathways such as the adrenergic signaling pathway, TGF-β signaling pathway, and glycerophospholipid metabolism. Among these, the enrichment in the glycerophospholipid metabolism pathway is closely associated with airway inflammation and lipid metabolism disorders in asthma. The STRING database was utilized to construct a PPI network of DEPs, which identified multiple hub proteins with strong connections. These proteins show correlation with inflammation regulation and airway remodeling related signatures. These correlational omics results support screening of candidate diagnostic biomarkers and hypothesis generation for targeted therapeutic development.

**Figure 3:**
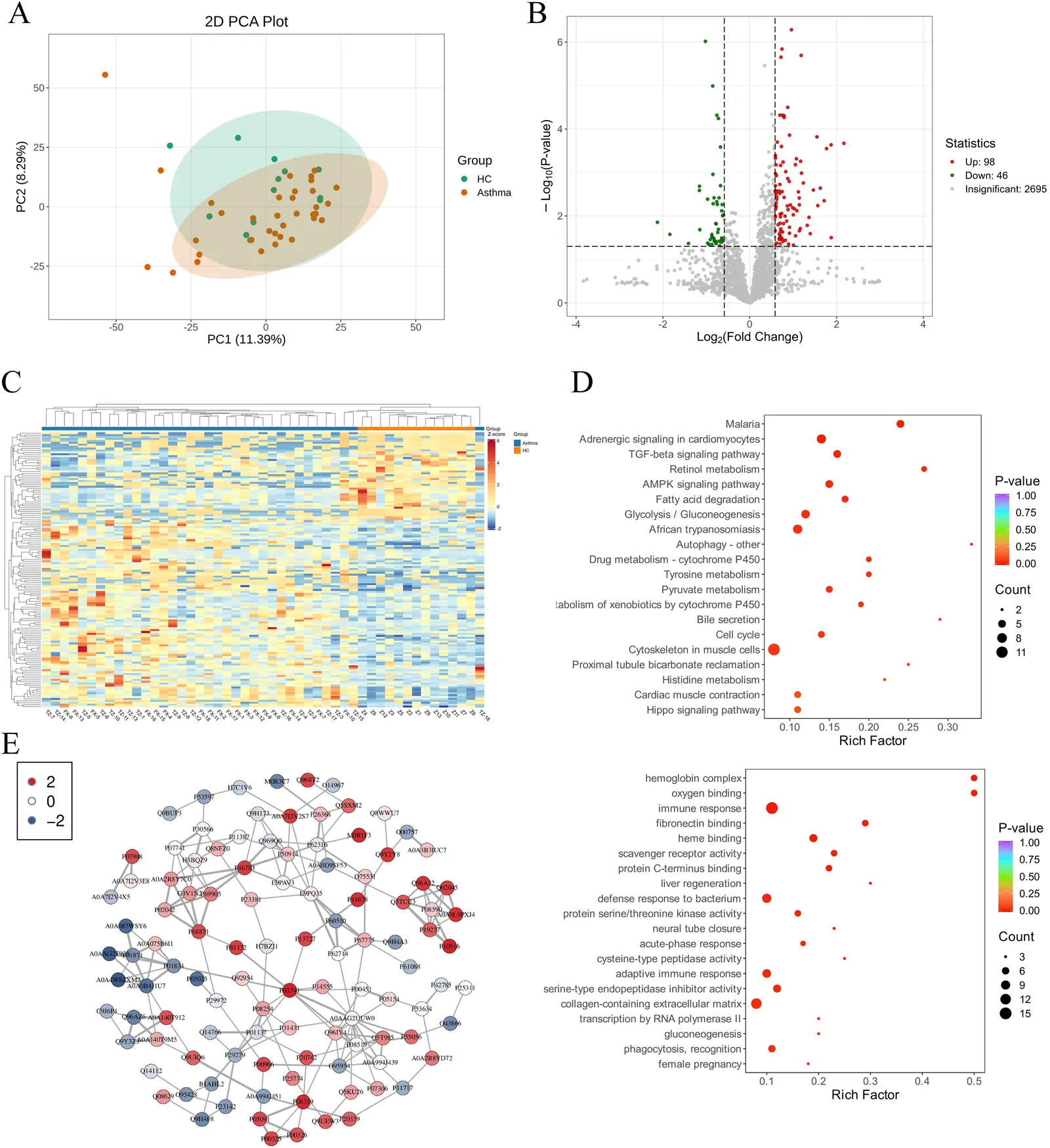
Proteomic Characteristics and Screening of Key Molecules and Pathways. **(A)** PCA plot showing the separation trend between asthma and healthy control samples. **(B)** In the volcano plot of differentially expressed proteins, red represents upregulated proteins, green represents downregulated proteins, and gray represents proteins without significant differences. **(C)** A heatmap of clustered proteins with differential expression, standardized using Z-scores, illustrating the variations in protein expression patterns between the two groups. **(D)** Bubble plots of GO functional enrichment and KEGG pathway enrichment for differentially expressed proteins, highlighting core enriched functional terms. **(E)** PPI network, where node colors represent expression changes (red for upregulation, blue for downregulation).

#### 2.2.4 Identification of differentially expressed metabolites in non-targeted metabolomics data between bronchial asthma patients and healthy controls (**Figures 4A–H**)

We performed comprehensive metabolomic analyses of serum samples from healthy controls and asthma patients to elucidate the mechanisms and changes associated with bronchial asthma at the metabolite level. Based on strict criteria of VIP > 1 and *P* < 0.05, 525 differentially expressed metabolites were identified, with 358 upregulated and 167 were downregulated, suggesting that overall metabolism in asthma exhibits a predominantly activated expression pattern. Analysis of differentially expressed metabolites identified 20 core compounds, of which 12 were significantly upregulated in the asthma group (e.g., triglycerides, bile acids, phospholipids, etc.), and 8 were significantly downregulated (intermediates of the tricarboxylic acid cycle and sphingolipids/phospholipids). Functional annotation of the differentially expressed metabolites using the KEGG database revealed that they are primarily associated with lipid and amino acid-related pathways, with lipid metabolism serving as the core module. The analysis of KEGG enrichment revealed essential metabolic pathways, including the biosynthesis of unsaturated fatty acids, steroid hormones, cholesterol metabolism, and the digestion and absorption of fats. Among these, the unsaturated fatty acid biosynthesis pathway showed the highest enrichment level, which was statistically significant. DA Score analysis showed that the DA Scores for the aforementioned core lipid metabolism pathways were all positive, indicating an overall trend of activation and upregulation in the asthma group. Once the matching information for the differentially expressed metabolites was obtained, pathway searches and regulatory interaction network analyses were performed using the KEGG database for the relevant species. The results, presented as network diagrams, suggest that these differentially expressed metabolites do not act independently but participate in the pathophysiological processes of asthma through synergistic interactions across multiple pathways. These differentially expressed metabolites and pathways yield candidate metabolite biomarkers and generate potential therapeutic target hypotheses for bronchial asthma.

**Figure 4:**
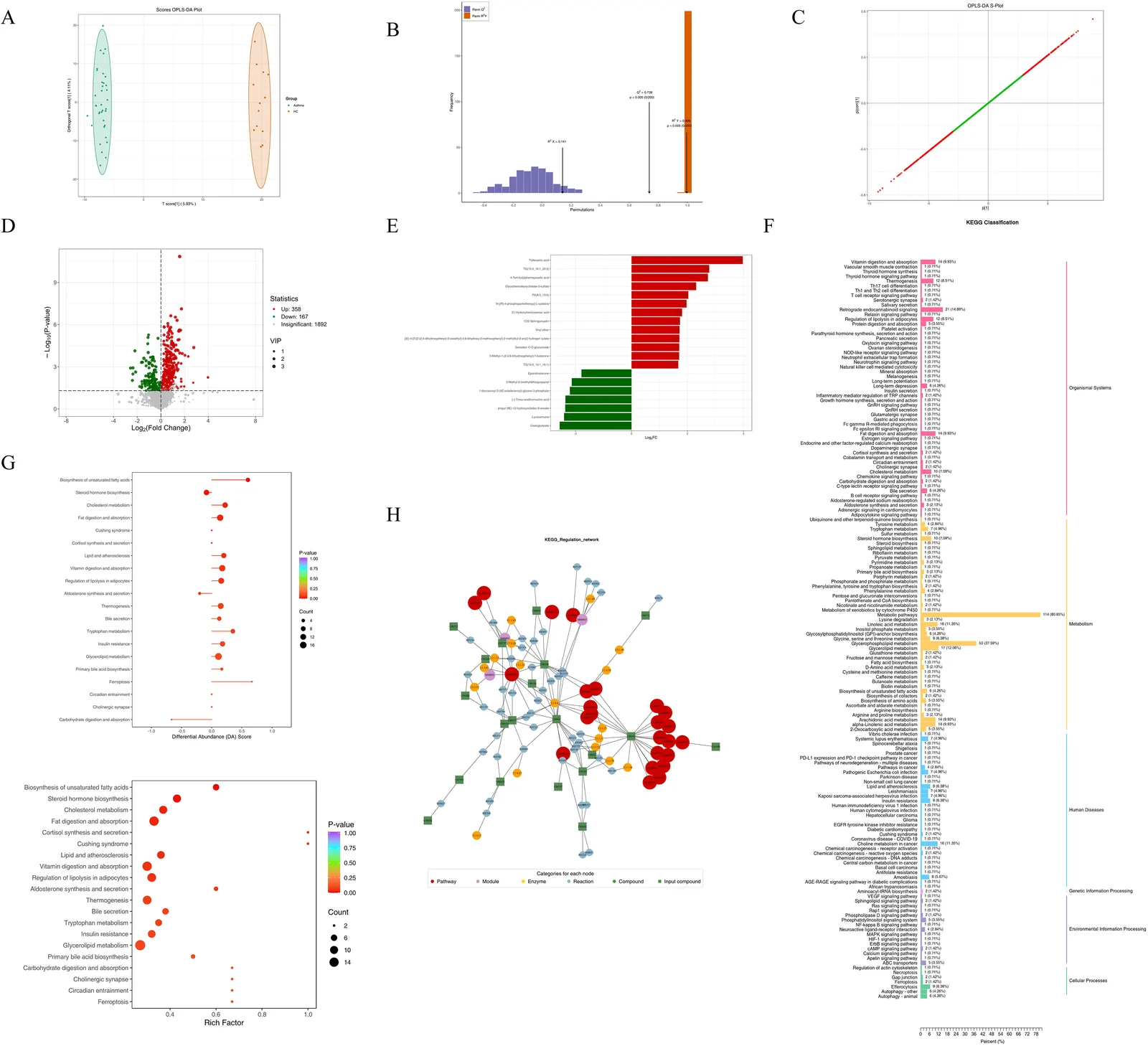
Metabolomic Characteristics of Asthma and Identification of Key Molecules and Pathways. **(A)** OPLS-DA score plot. **(B)** OPLS-DA replacement test plot. **(C)** OPLS-DA S-plot. **(D)** Volcano plot of differentially expressed metabolites. **(E)** Bar chart of differentially expressed metabolites. **(F)** Classification plot of differentially expressed metabolite pathways. **(G)** Enrichment plot and abundance score plot of differentially expressed metabolite pathways. **(H)** Regulatory network plot of differentially expressed metabolites.

#### 2.2.5 Identification of differentially abundant microbial species in oral microbiome between bronchial asthma patients and healthy control groups (**Figures 5A–H**)

This study systematically analyzed the oral microbiome characteristics of the Asthma and HC groups. First, Venn diagram analysis revealed that 653 shared OTUs were detected across both groups, with 146 OTUs specific to the Asthma group and 27 OTUs specific to the HC group, suggesting that the microbiome composition of the Asthma group exhibits higher specificity. Alpha diversity analysis showed that there were no significant statistical differences in the Chao1, Shannon indices (*P* > 0.05), indicating no significant changes in microbial richness or diversity between the two groups. In β-diversity analysis, PCoA analysis revealed significant differences in microbial community structure between the two groups (Adonis R² = 0.06, *P* = 0.014), and NMDS analysis (stress = 0.22) further validated this separation trend, confirming characteristic alterations in microbial community structure among asthma patients. Analysis of microbial composition at the phylum level showed that both groups were dominated by the Proteobacteria, Firmicutes, and Bacteroidetes phyla, Significant differences in the relative abundances of core genera, including Haemophilus and Streptococcus, were revealed between the two groups at the genus level. LEfSe analysis identified characteristic microbial communities significantly enriched in the HC group, including Haemophilus parainfluenzae, Porphyromonas gingivalis, the Haemophilus genus, the Pasteurellaceae family, and the Enterobacteriaceae family. Among these, Haemophilus parainfluenzae had the highest LDA score and was identified as the signature differential bacterium for the HC group. The two groups exhibited significant differences in microbial function, as revealed by functional prediction analysis. with the HC group showing significant enrichment in pathways such as energy metabolism, lipopolysaccharide biosynthesis proteins, and the sulfur relay system, whereas the Asthma group was enriched in pathways including neomycin, kanamycin, and gentamicin biosynthesis, as well as bacterial toxins. This suggests significant differences between the two groups in terms of metabolic function and immune regulatory capacity, providing a molecular basis for research into the Oral microbiome-immune regulatory mechanisms of asthma.

**Figure 5:**
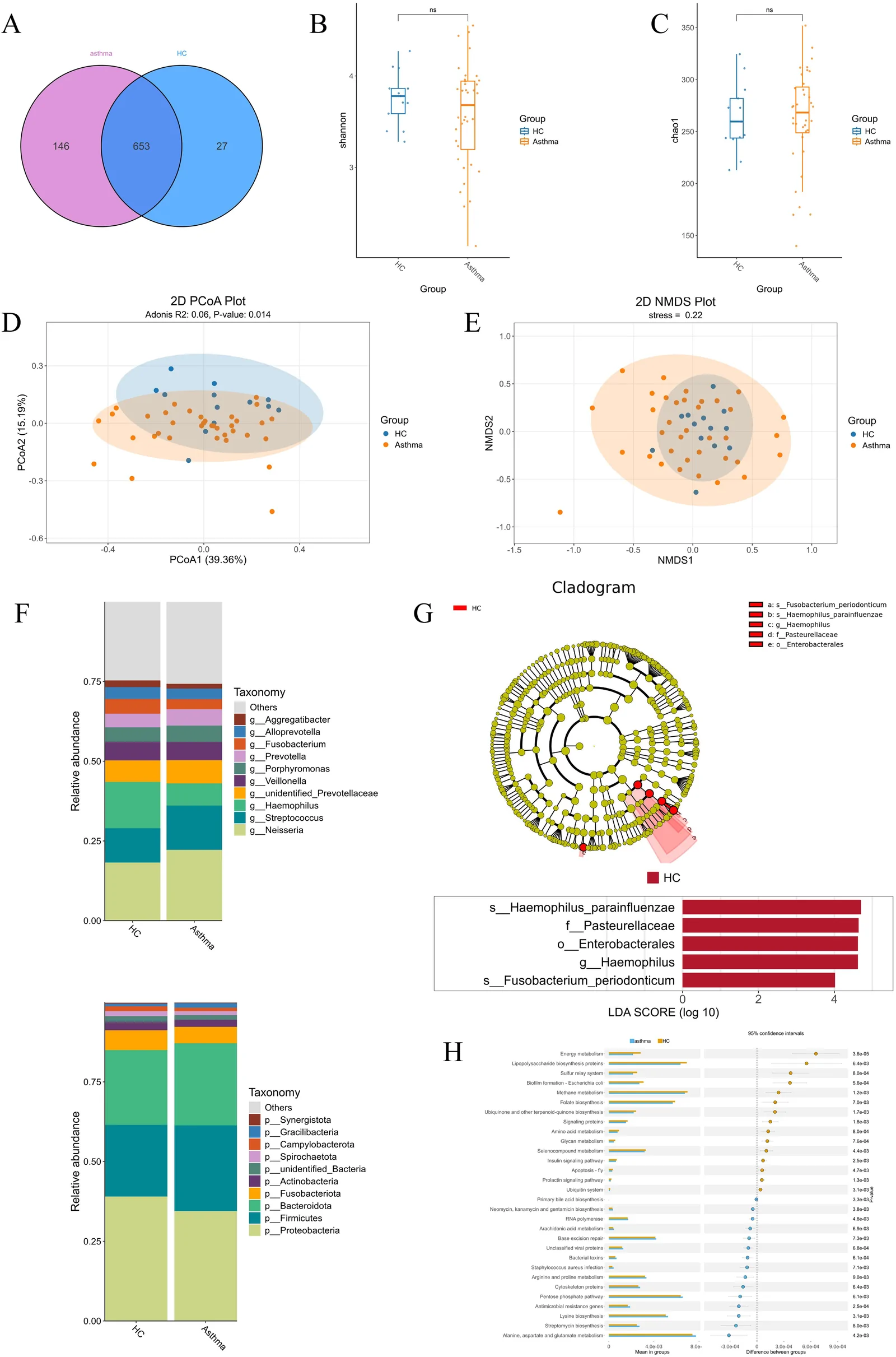
Analysis of Oral Microbiome Characteristics and Differences in Asthma Patients. **(A)** The Venn diagram displays the quantity of microbial species that overlap and differ between individuals with asthma and those in the healthy control group. **(B)** Box plot of Shannon index α-diversity, comparing differences in oral microbiome diversity between the two groups. **(C)** Box plot of Chao1 index α-diversity, comparing differences in oral microbiome richness between the two groups. **(D)** β-diversity PCoA plot, showing the separation trends in microbial community structure between the two groups based on Bray-Curtis distance. **(E)** NMDS plot, illustrating differences in oral microbiota structure between the two groups. **(F)** Stacked bar chart of microbial composition at the phylum/genus level, illustrating structural differences in oral microbiome between the two groups. **(G)** LEfSe phylogenetic tree and LDA score bar chart, identifying significantly different characteristic microbial communities between the two groups. **(H)** Microbial functional difference analysis, showing significantly different KEGG functional pathways between the two groups.

#### 2.2.6 Identification of differentially expressed genes between the Lung Qi Deficiency group and the Phlegm-Dampness Obstructing the Lungs group in transcriptomics (**Figures 6A–G**)

PCA analysis showed that the first two principal components collectively explained 22.33% of the total variance. LQD and PZLP samples exhibited a highly overlapping distribution in the two-dimensional space, suggesting that there were no significant differences in the overall transcriptome between the two asthma patterns. The analysis of differential expression revealed 401 significant DEGs, with 263 showing upregulation and 138 showing downregulation in the LQD group versus the PZLP group. Core DEGs such as OR7D2, IL1A, and LUM were significantly upregulated in the LQD group, while genes such as CTXN2-AS1 were downregulated. GO enrichment analysis indicated that DEGs were significantly enriched in biological processes such as bacterial molecular responses, leukocyte migration, and humoral immunity against bacteria. At the cellular component level, they were enriched in secretory granules and cytoplasmic vesicles; at the molecular function level, they were enriched in cytokine activity and receptor-ligand interactions. KEGG pathway enrichment further confirmed that DEGs were significantly enriched in cytokine-cytokine receptor interactions, chemokine signaling pathways, and the IL-17 signaling pathway, while also involving disease pathways such as fluid shear stress and atherosclerosis. WGCNA analysis revealed that the red, green, light-yellow, yellow, and purple modules were significantly positively correlated with the PZLP phenotype, while showing negative correlation with the LQD phenotype. By contrast, the midnight-blue module displayed the opposite correlation pattern, suggesting specific co-expression network reorganization among different asthma TCM subtypes. PPI network analysis further highlighted MMP9 as a central hub gene linking immune-inflammatory and extracellular matrix remodeling pathways, which may play a key role in the molecular differences between the LQD and PZLP patterns. In summary, this study systematically identifies transcriptomic differences between LQD and PZLP centered on immune inflammatory and tissue-remodeling associated signatures. These correlational results support generating mechanistic hypotheses for TCM asthma patterns.

**Figure 6:**
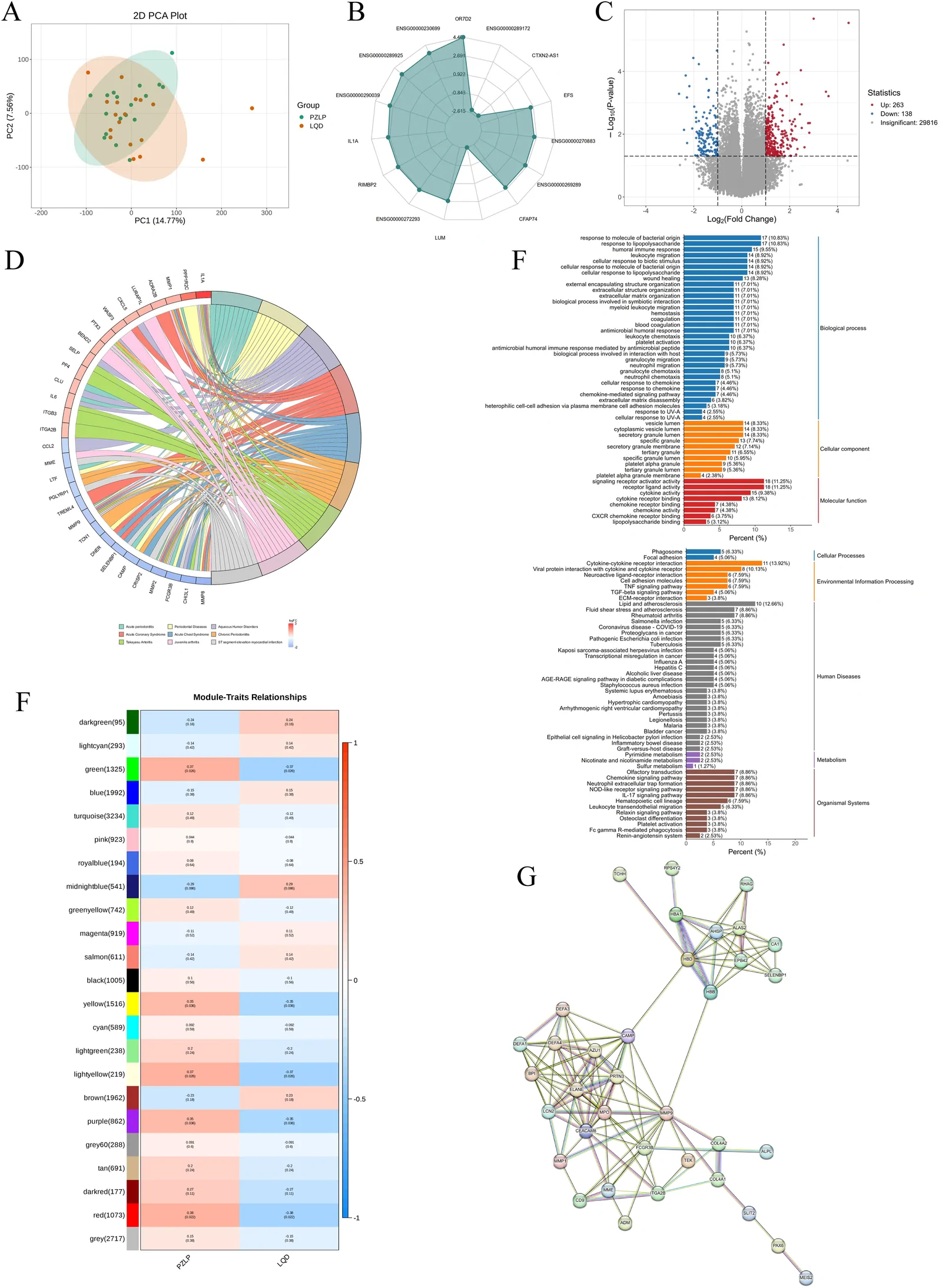
Transcriptomic Characteristics of LQD and PZLP and Screening of Key Molecules and Pathways. **(A)** PCA plot of transcriptomic data from LQD and PZLP. **(B)** Radar plot of core differentially expressed gene characteristics. **(C)** Genes with differential expression are displayed in the volcano plot: red signifies upregulation, blue signifies downregulation, and gray signifies no significant difference. **(D)** Circle plot of association analysis for asthma-related phenotypes. **(E)** Genes with differential expression underwent GO functional and KEGG pathway enrichment analysis. **(F)** Heatmap showing the correlation between gene co-expression clusters and disease phenotypes. Rows represent different WGCNA clusters, columns represent the PZLP and LQD groups, and colors indicate correlation coefficients (red for positive correlation, blue for negative correlation). **(G)** PPI network illustrating the interaction relationships among key genes and core regulatory modules between the two groups.

#### 2.2.7 Identification of differentially expressed proteins between the Lung Qi Deficiency group and the Phlegm-Dampness Obstructing the Lungs group via proteomics (**Figures 7A–F**)

Patients with bronchial asthma exhibiting different inflammatory phenotypes demonstrate significant heterogeneity in their clinical treatment responses. Clearly distinguishing between the LQD and PZLP patterns is crucial for optimizing clinical management strategies; however, the underlying proteomic regulatory mechanisms remain incompletely elucidated. Through proteomic analysis, this study systematically elucidated the molecular profiles of the LQD and PZLP patterns and explored their interacting biological networks, aiming to establish phenotype-specific diagnostic and therapeutic biomarkers. Hierarchical clustering heatmap analysis revealed a distinct separation in the expression profiles of LQD and PZLP samples, with characteristic differentially expressed patterns between the two groups. Certain proteins were significantly upregulated in the LQD group, while the PZLP group exhibited opposite expression patterns, suggesting significant molecular expression differences between the two groups; PCA analysis further validated the grouping characteristics of the samples. PC1 and PC2 explained 13.59% and 9.29% of the total variance, respectively. The samples from both groups showed partial clustering with a small number of outliers, as visually observed in the PCA plot. Based on the screening criteria of |log_2_(Fold Change)| > 1 and *P* < 0.05, a total of 44 DEPs were identified, including 25 proteins significantly upregulated in the LQD group and 19 proteins significantly downregulated, thereby defining the core set of differentially expressed molecules between the two groups. Comprehensive GO and KEGG enrichment analyses revealed that DEPs were significantly enriched in core biological processes such as ATP binding, ATP hydrolysis, MAPK signaling, and misfolded protein binding. Meanwhile, KEGG pathway analysis further revealed that immune and inflammatory-related pathways—such as FcϵRI, Toll-like receptors, TNF, and MAPK signaling—as well as metabolic pathways including pyrimidine metabolism and cysteine and methionine metabolism were significantly enriched between the two groups. This confirms the key regulatory roles of dysregulated immune responses and metabolic abnormalities in the pathophysiological differences between the two groups. Protein interaction network analysis established regulatory relationships among differentially expressed proteins. Within the network, upregulated and downregulated proteins formed tightly connected core modules; Certain core proteins with high connectivity might be crucial in regulating the pathophysiological differences between the two groups, offering a central focus for future mechanistic research and target discovery.

**Figure 7:**
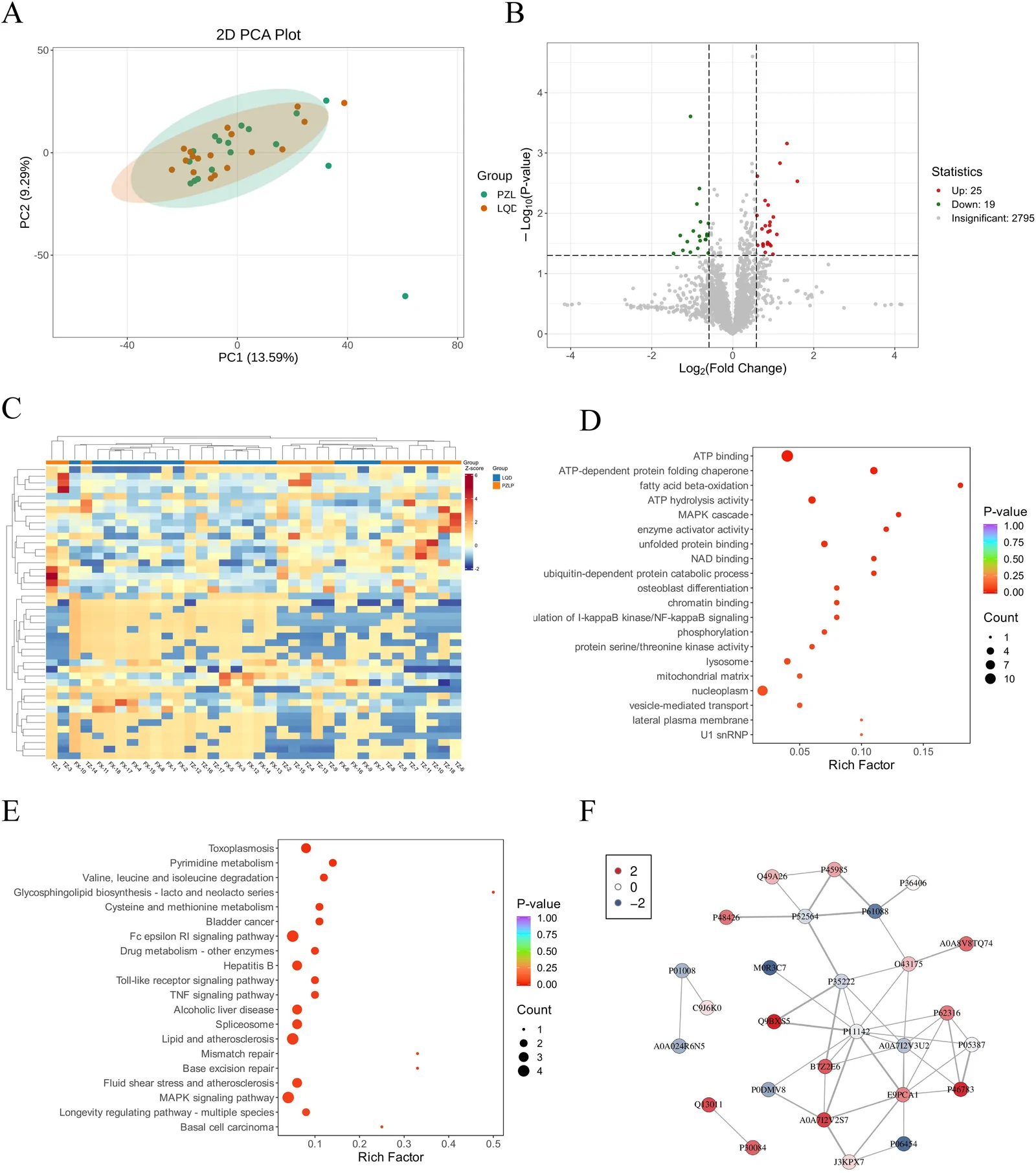
Proteomic Characteristics of LQD and PZLP and Screening of Key Molecules and Pathways. **(A)** PCA plot showing the separation trends between LQD and PZLP samples. **(B)** Volcano plot of differentially expressed proteins: red indicates upregulated proteins, green indicates downregulated proteins, and gray indicates proteins with no significant difference. **(C)** Clustering heatmap of differentially expressed proteins, standardized by Z-score, showing differences in protein expression patterns between the two groups. **(D)** GO analysis for functional enrichment of proteins with differential expression. **(E)** KEGG pathway enrichment bubble plot, showing core enriched functional terms. **(F)** PPI network, where node colors represent expression changes (red for upregulation, blue for downregulation).

#### 2.2.8 Identification of differentially expressed metabolites in the Lung Qi Deficiency Group and the Phlegm-Dampness Obstructing the Lungs Group via non-targeted metabolomics (**Figures 8A–H**)

We performed metabolomic analysis on serum samples from asthma patients with PZLP and LQD patterns and identified 237 differentially expressed metabolites based on the aforementioned criteria, including 79 upregulated and 158 downregulated metabolites. When compared to the LQD group, the PZLP group showed the highest upregulation in metabolites like carnosol, phospholipid PE (16:1,20:0), and p-cresol, among other lipids and phenolic compounds, whereas the Lung Qi Deficiency group showed significant upregulation of 1-stearoyl-2-arachidonylglycerol, PE-NMe2 (20:2_20:2), urobilinogen, and other glycerol esters, bile acids, and neuroactive metabolites. Functional annotation of differentially expressed metabolites between the two groups using the KEGG database revealed that the differentially expressed metabolites in asthma patients were primarily associated with pathways related to lipid metabolism, amino acid metabolism, and bile acid metabolism, accompanied by synergistic abnormalities in the nervous and autophagy systems. The PZLP group showed a notable increase in pathways associated with glycerophospholipid metabolism, sphingolipid metabolism, retrograde endocannabinoid signaling, and autophagy compared to the LQD group; conversely, fatty acid degradation pathways were more active in the Lung Qi Deficiency group, with glycerophospholipid metabolism showing the highest enrichment and the most significant difference; The core metabolic differences between the LQD and PZLP patterns manifest as a bidirectional imbalance in lipid metabolism pathways. By visualizing the correlations among multidimensional elements—including pathways, modules, enzymes, reactions, and compounds—we demonstrate the pathway-enzyme-reaction regulatory network involving differential metabolites, thereby revealing the systemic mechanisms underlying metabolic disorders. The differences in metabolic dysregulation between the two groups are not isolated pathway abnormalities, but rather systemic network abnormalities characterized by synergistic dysregulation across multiple pathways, enzymes, and reactions, with lipid metabolism at the core. These correlational multi-metabolite findings provide a global perspective for generating mechanistic hypotheses and supply metabolomic candidate signatures for TCM syndrome objectification research.

**Figure 8:**
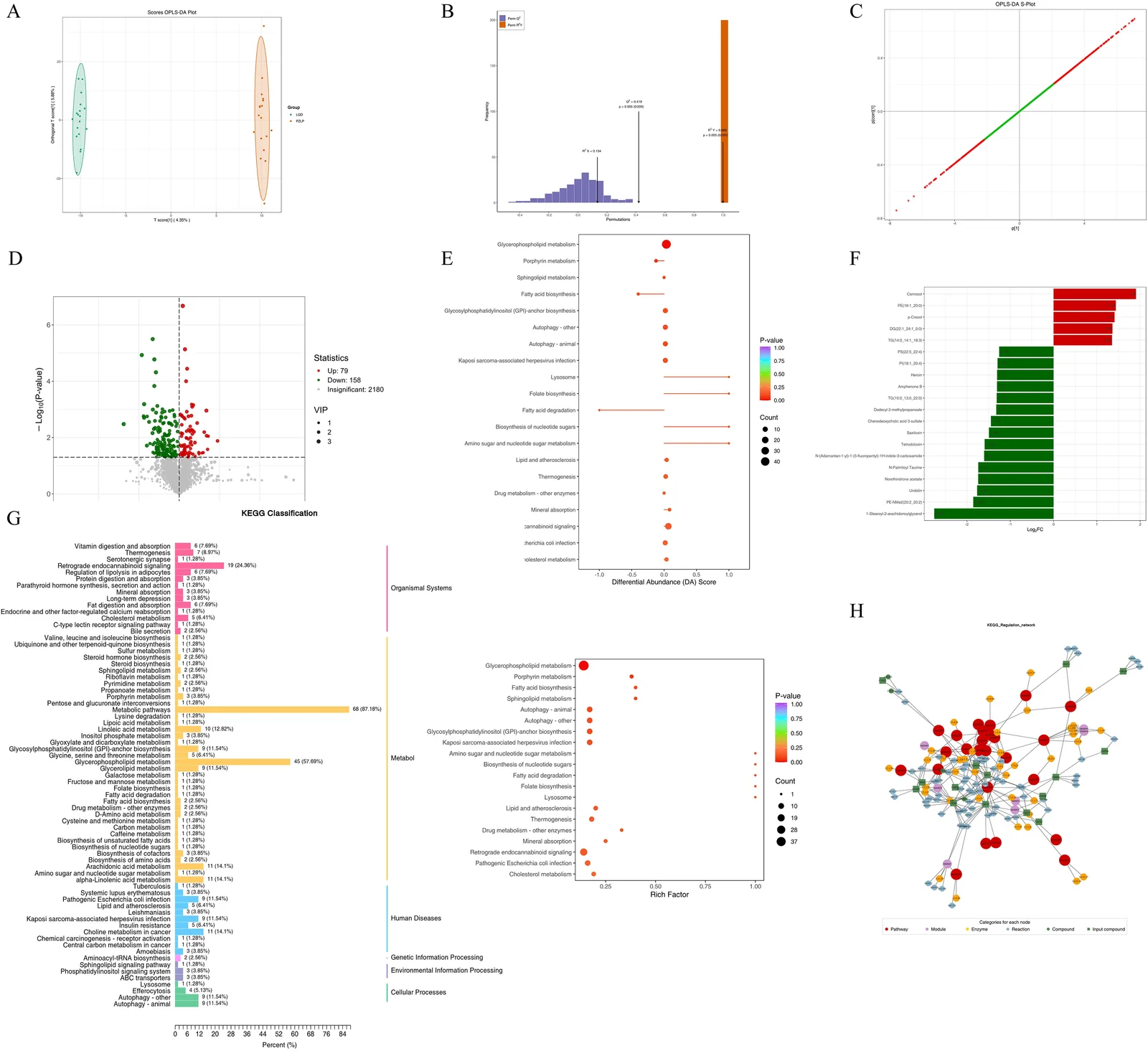
Metabolomic profiles of LQD and PZLP, and screening of key molecules and pathways. **(A)** OPLS-DA score plot. **(B)** OPLS-DA replacement test plot. **(C)** OPLS-DA S-plot. **(D)** Volcano plot of differentially expressed metabolites. **(E)** Bar chart of differentially expressed metabolites. **(F)** Classification plot of differentially expressed metabolite pathways. **(G)** Enrichment plot and abundance score plot of differentially expressed metabolite pathways. **(H)** Regulatory network plot of differentially expressed metabolites.

#### 2.2.9 Identification of differential microbial communities in oral between the Lung Qi Deficiency Group and the Phlegm-Dampness Obstructing the Lungs group (**Figures 9A–G**)

This investigation analyzed the structural and functional disparities in the oral microbiome between the LQD and PZLP patterns in a systematic manner. Venn diagram analysis revealed that 653 shared OTUs were detected in both groups, with 96 OTUs specific to the LQD group, while the PZLP group had 50 unique OTUs, suggesting that the microbial composition of the LQD group exhibits higher specificity. The analysis of α-diversity further validated that the Chao-1 index was not statistically different between the two groups (*P* > 0.05), and the Shannon indices of the two groups showed no significant statistical difference (*P* > 0.05), indicating that the overall diversity levels of the microbiota in both groups did not undergo significant changes. PCoA analysis in β-diversity revealed no notable separation trend in the microbial structures between the two groups (Adonis R² = 0.03,

**Figure 9:**
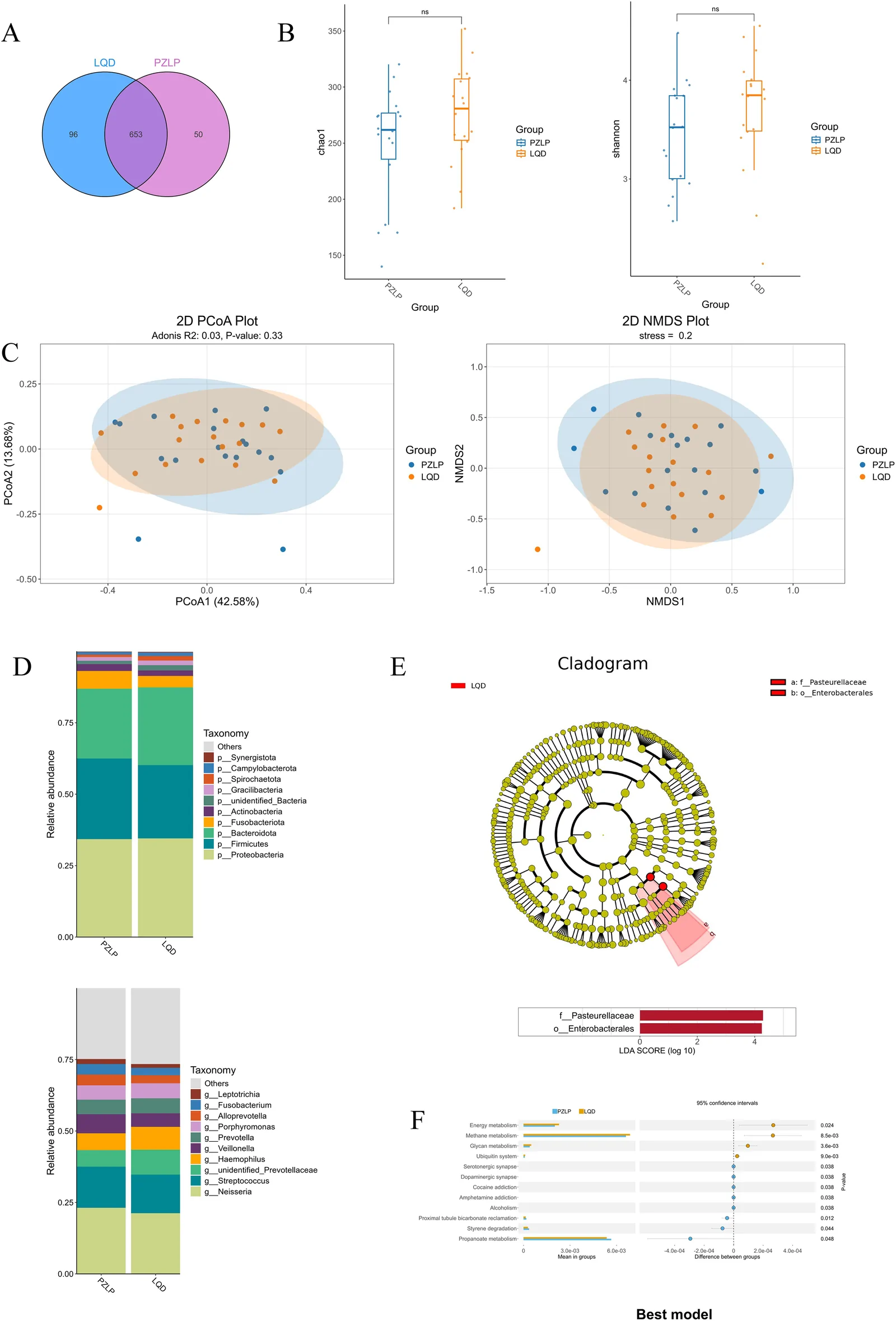
Oral microbiome characteristics and differential analysis between LQD and PZLP groups. **(A)** Venn diagram illustrating the count of common and distinct microbial taxa between the asthma group and the healthy control group. **(B)** Comparative box plots of α-diversity (Chao-1 and Shannon indices) showing differences in microbial richness and diversity between the two groups. **(C)** β-diversity PCoA and NMDS plots, illustrating the separation trends in microbial community structure between the two groups. **(D)** Stacked bar charts of microbial composition at the phylum/genus levels, demonstrating differences in microbial community structure between the two groups. **(E)** LEfSe phylogenetic tree and LDA score bar charts, identifying characteristic microbial communities with significant differences between groups. **(F)** ROC curve of the optimal model (AUC = 0.889), demonstrating the model’s diagnostic performance in distinguishing between the two patient groups; analysis of functional differences in oral microbiome between the two groups, showing KEGG functional pathways with significant differences between the PZLP and LQD groups. **(G)** Ranking of species importance for oral microbiome differences between PZLP and LQD asthma patients.

= 0.33), and NMDS analysis (stress = 0.2) also verified the similarity of the overall microbial structures between the two groups. At the phylum level, both groups were dominated by Proteobacteria, Firmicutes, and Bacteroidetes. At the genus level, differences were observed in the abundance of core genera such as Haemophilus and Streptococcus. LEfSe analysis identified the Pasteurellaceae and Enterobacteriaceae families, which were significantly enriched in the LQD group, as signature differential taxa. Random Forest analysis identified Bacillus and Cardiobacterium as potential high-importance typing markers. The diagnostic model constructed based on these characteristic bacteria achieved an AUC of 0.889 (95% CI: 0.786–0.992), demonstrating excellent typing predictive performance; Analysis of functional predictions showed notable differences in microbial functional profiles between the two groups. The LQD group was significantly enriched in pathways such as methane metabolism, glycerol metabolism, and the ubiquitin system, while the PZLP group was enriched in pathways including energy metabolism, serotonergic synapses, dopaminergic synapses, and cocaine addiction. The differences in metabolic and neuro-signaling-related functions between the two groups suggest that they may participate in host physiological regulation through distinct microbial metabolic pathways. These correlational microbiome findings provide candidate microbial signatures and generate theoretical hypotheses for precise differentiation of LQD and PZLP patterns, phenotypic classification, and further investigation of host microbiota interaction mechanisms.

#### 2.2.10 Multiomics integrated analysis of patients with bronchial asthma and healthy controls (**Figures 10A–M**)

This investigation employed transcriptomic, proteomic, and metabolomic data to systematically explore the molecular disparities between asthma patients and HC, comprehensively revealing the multidimensional molecular regulatory network underlying asthma pathogenesis. Joint transcriptomic and proteomic analysis identified four common differentially expressed molecules across both omics datasets. Correlation analysis indicated low consistency in expression trends between the two omics (R² = 0.003). These differentially expressed molecules were found to be jointly enriched in biological processes, including immune defense and bacterial response, according to GO enrichment analysis, while KEGG pathway analysis showed common enrichment in inflammation-related pathways including TNF, IL-17, PI3K-Akt, and other inflammation-related pathways. The protein interaction network identified core regulatory modules, confirming MMP9 and the DEFA family as key hub molecules. Integrated transcriptomic and metabolomic analysis revealed that hierarchical clustering heatmaps confirmed significant separation between the metabolite expression profiles of the asthma group and the control group. Both omics datasets were jointly enriched in inflammation- and metabolism-related pathways, such as arachidonic acid metabolism, chemokine signaling, and neutrophil extracellular trap formation. A metabolite-gene interaction network was constructed to identify core regulatory modules and key regulatory molecules. Combined proteomic and metabolomic analysis revealed that hierarchical clustering heatmaps validated the significant separation of metabolic profiles between the two groups. Joint KEGG pathway enrichment analysis indicated that adrenergic signaling and cytochrome P450 metabolism were significantly enriched pathways common to both omics datasets, while the metabolite-protein interaction network identified core hub molecules. This integrated multi-omics analysis systematically identifies correlational molecular networks at transcriptional, proteomic and metabolic levels, furnishing an important basis to generate hypotheses for precise diagnosis, treatment and target screening.

**Figure 10:**
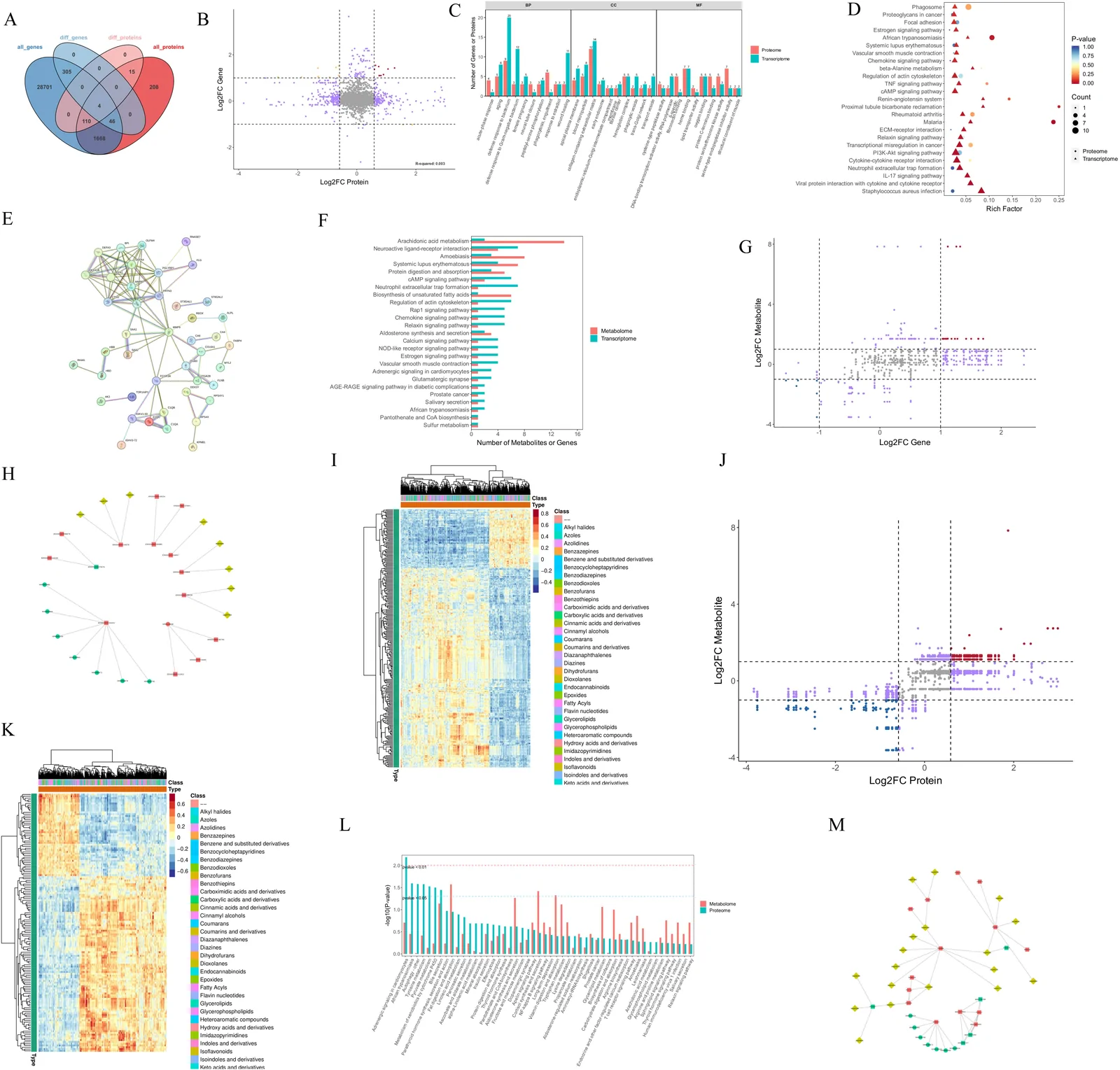
Multiomics integrated analysis of patients with bronchial asthma and healthy controls. **(A–E)** Integration of transcriptomics and proteomics; **(F–I)** Integration of transcriptomics and metabolomics; **(J–M)** Integration of proteomics and metabolomics. **(A)** Venn diagram showing the number and overlap of differentially expressed molecules across the two omics. **(B)** Scatter plot of expression fold changes, showing the correlation between expression changes across the two omics; **(C)** GO functional enrichment bar chart, showing core functions co-enriched across the two omics; **(D)** KEGG pathway enrichment bubble plot, showing core pathways co-enriched across the two omics. **(E)** PPI network of co-differentially expressed molecules, showing core regulatory modules and targets. **(F)** The analysis of KEGG pathways showed that core regulatory pathways are enriched in both transcriptomic and metabolomic data. **(G)** Scatter plot of the correlation between transcriptomic and metabolomic fold changes, showing the correlation between expression changes across both omics. **(H)** Clustering heatmap of differentially expressed metabolites, illustrating differences in metabolic expression patterns between groups. **(I)** Gene-metabolite association regulatory network, illustrating the interactions between core differentially expressed genes and metabolites. **(J)** Scatter plot of the correlation between proteomic and metabolomic expression fold changes, illustrating the correlation between changes in these two omics profiles. **(K)** Clustering heatmap of differentially expressed metabolites, illustrating differences in metabolic expression patterns between groups. **(L)** Enrichment analysis of differentially expressed molecular pathways in proteomics and metabolomics, showing core regulatory pathways co-enriched across both omics. **(M)** Protein-metabolite association regulatory network, showing the interactions between core differentially expressed proteins and metabolites.

#### 2.2.11 Multiomics integrated analysis of the PZLP and LQD groups (**Figures 11A–M**)

The research combined transcriptomic, proteomic, and metabolomic data to thoroughly examine the molecular distinctions between the PZLP and LQD groups, uncovering the complex molecular regulatory networks of these two TCM syndromes. Joint transcriptomic and proteomic analysis identified 75 common differentially expressed molecules; correlation analysis indicated extremely low consistency in expression trends between the two omics (R² = 0.000). Joint GO and KEGG enrichment analysis revealed that both omics datasets were enriched in inflammation-related pathways such as chemokines and TNF, as well as biological processes related to immune defense. The construction of a protein interaction network identified core regulatory modules, clarifying that the MMP family and the keratin family are key hub molecules. Combined proteomics and metabolomics analysis revealed that hierarchical clustering heatmaps confirmed significant separation in the metabolite expression profiles between the two phenotypes. Both omics were jointly enriched in metabolic pathways, lipid metabolism, and atherosclerosis-related pathways, and the metabolite-protein interaction network identified core regulatory molecules. Combined transcriptomic and metabolomic analysis revealed that hierarchical clustering heatmaps validated the significant separation of metabolic profiles between the two subtypes. Joint KEGG pathway enrichment analysis indicated that glycerophospholipid metabolism, lipids, and atherosclerosis were common enriched pathways across both omics, and the metabolite-gene interaction network identified core regulatory modules. Multiomics integrated analysis systematically elucidated the molecular regulatory mechanisms of the two syndrome patterns at the transcriptional, proteomic, and metabolic levels, providing a crucial theoretical basis for TCM syndrome differentiation, mechanism elucidation, and precision diagnosis and treatment.

**Figure 11:**
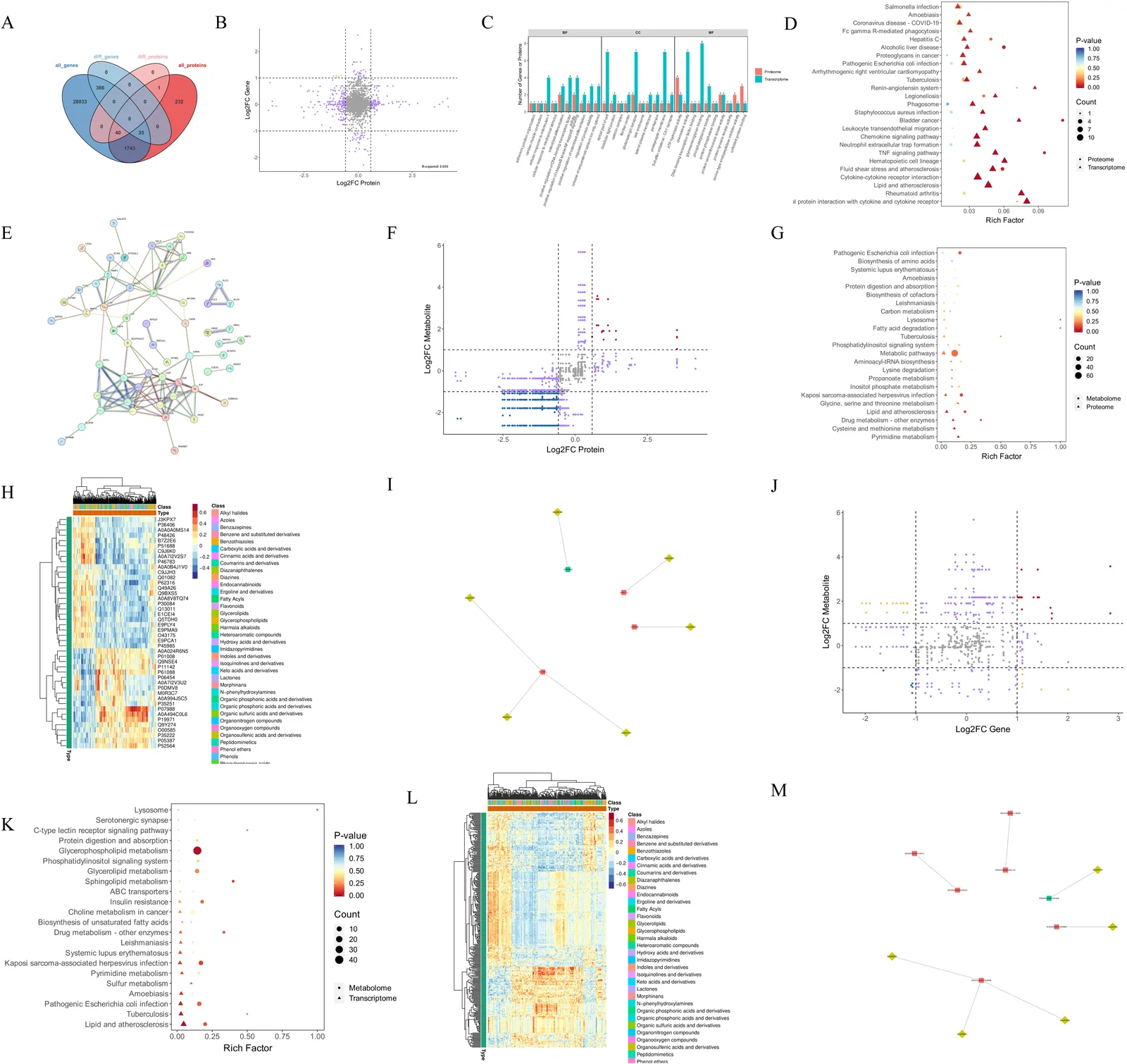
Multiomics integrated analysis of the Phlegm-Dampness Obstructing the Lungs group and the Lung Qi Deficiency group. **(A–E)** Integrated transcriptomics and proteomics; **(F–I)** Integrated proteomics and metabolomics; **(J–M)** Integrated transcriptomics and metabolomics. **(A)** Venn diagram showing the number and overlap of differentially expressed molecules across the two omics; **(B)** Expression fold change correlation scatter plot showing the correlation between expression changes across the two omics; **(C)** GO functional enrichment bar chart showing core functions co-enriched across the two omics; **(D)** KEGG pathway enrichment bubble plot showing core pathways co-enriched across the two omics; **(E)** PPI network of co-differentially expressed molecules, showing core regulatory modules and targets; **(F)** Scatter plot of proteomic and metabolomic fold-change correlations, illustrating the correlation between expression changes across the two omics; **(G)** Pathway enrichment analysis of differentially expressed molecules in proteomics and metabolomics, highlighting core regulatory pathways co-enriched across the two omics; **(H)** Clustering heatmap of differentially expressed metabolites, illustrating differences in metabolic expression patterns between groups. **(I)** Protein-metabolite association regulatory network, illustrating the interaction relationships between core differentially expressed proteins and metabolites. **(J)** Scatter plot of the correlation between transcriptomic and metabolomic expression fold changes, demonstrating the correlation between changes in the two omics profiles. **(K)** Enrichment analysis of differentially expressed molecular KEGG pathways in the transcriptome and metabolome, showing core regulatory pathways co-enriched in both omics. **(L)** Clustering heatmap of differentially expressed metabolites, showing differences in metabolic expression patterns between groups. **(M)** Gene-metabolite association regulatory network, showing the interactions between core differentially expressed genes and metabolites.

### 2.3 Discussion

The heterogeneity of bronchial asthma represents a significant challenge that impedes its accurate diagnosis and treatment (20). The TCM concept of “same disease with different syndromes,” which originates from the Huangdi Neijing, is central to the system of syndrome differentiation and treatment. This concept posits that the same disease can manifest in different syndromes due to variations in patients’ constitutions, disease progression, and pathogeneses (21). This theoretical framework aligns closely with the contemporary medical understanding of “disease heterogeneity,” offering a distinctive perspective for analyzing asthma heterogeneity. However, the molecular basis and microecological regulatory mechanisms underlying the two primary asthma syndromes, Phlegm-Dampness Obstructing PZLP and LQD, remain inadequately explored (22). Current multi-omics research on asthma predominantly examines molecular differences between asthmatic patients and healthy individuals or focuses solely on comparing molecular profiles of distinct inflammatory endotypes. Few studies have successfully integrated transcriptomic, proteomic, metabolomic, and microecological data through the lens of TCM syndromes, with even fewer investigations systematically generating hypotheses regarding functional regulatory discrepancies underlying the phenomenon of “same disease with different syndromes” (23–25). In this study, we employed multi-omics technologies to systematically construct, for the first time, a comprehensive multi-dimensional molecular signature landscape of asthma PZLP and LQD syndromes. Our findings suggest that a “bidirectional imbalance of lipid metabolism-microbiota” may serve as the core molecular discriminative feature distinguishing these two syndromes. Furthermore, we suggest that the functional differentiation of oral microbiota plays a crucial role in syndrome formation, providing a novel correlational perspective for interpreting the pathological nature of bronchial asthma through the dimension of syndrome heterogeneity (26).

This study provides correlational omics signatures suggestive of disrupted glycerophospholipid metabolism, potential excessive activation of the NF-κB inflammatory pathway, and impaired phagosome function collectively form a critical pathological axis in the onset of asthma. This finding not only corroborates the classical theory proposed by Kume et al. (27), which posits that lipid mediators exacerbate inflammation in severe asthma, but also broadens the conventional understanding of abnormal lipid metabolism beyond the arachidonic acid pathway to include the glycerophospholipid metabolic network. Mechanistically, altered expression of PLD5 observed in our dataset is hypothesized to be a pivotal upstream event linked to glycerophospholipid metabolic disturbances. As a member of the phospholipase D family, reduced PLD5 expression is correlatively associated with disturbed glycerophospholipid catabolism, resulting in abnormal accumulation of pro-inflammatory lipid related metabolites in serum such as sphingomyelin and phosphatidylcholine (28, 29). On a hypothetical mechanistic level, these lipid mediators might directly disrupt airway epithelial cell membrane integrity and may trigger cascade release of pro-inflammatory factors including IL-1β and TNF-α via the TNFRSF10C-NF-κB signaling axis (30). Meanwhile, lipid accumulation hypothetically could inhibit phagosome maturation and lysosomal acidification in macrophages (31, 32), resulting in impaired clearance of apoptotic cells and inflammatory debris, thus forming a hypothesized vicious pathological cycle of “lipid accumulation-inflammatory activation-dysfunctional phagocytosis”. This proposed mechanistic chain provides new testable hypotheses for understanding how lipid-metabolism perturbation may drive chronic asthmatic inflammation; nevertheless, direct cell-based or animal-model functional validation is still required. Notably, the consistency of transcriptomic and proteomic expression trends in this study was extremely low (R²=0.003 for overall asthma vs healthy controls; R²<0.0001 for inter-syndrome comparisons). Relevant studies have confirmed that pulmonary inflammatory stimulation decouples transcription and protein expression of immune molecules (33, 34). These published reports suggest several plausible underlying mechanisms that may account for this transcript-protein discordance, including translational regulation of lipid metabolic genes mediated by m^6^A epigenetic modification (26, 35), transcriptional silencing mediated by miRNAs and stress granules (36), as well as selective protein degradation via the ubiquitin-proteasome system (37). We emphasize that our present dataset cannot formally prove that these post-transcriptional processes directly produce the low concordance observed herein; instead, these well-documented pathways offer potential biological rationality for the post-transcriptional reprogramming frequently seen under inflammatory conditions. Such post-transcriptional reprogramming itself represents a core pathological phenotype of asthma, and transcriptomic data alone cannot reflect actual functional status, highlighting the necessity of integrated multi-omics analysis. Significant bidirectional imbalance of lipid metabolism featuring “excessive lipid synthesis vs impaired lipid oxidation” was identified between the two syndromes, providing correlational molecular clues for asthmatic syndrome heterogeneity (38).

A pivotal correlational finding of this study is that PZLP and LQD syndromes display distinct omics-derived molecular signatures suggestive of bidirectional lipid-metabolic imbalance, rather than merely differences in inflammatory severity as previously recognized. Essentially, the two syndromes represent two clinical subtypes with unique correlational molecular signatures under the background of asthmatic heterogeneity. For the PZLP syndrome, our data show molecular signatures hypothetically linked to SREBP-1c-mediated excessive lipogenesis (39), accompanied by correlational aberrant MAPK signaling-pathway signatures and accumulation of pro-inflammatory lipid metabolites in serum (40). Hypothetically, dysregulated lipid metabolism may induce hyperplasia of airway epithelial cells and goblet cells, further leading to airway mucus hypersecretion (41). This molecular phenotype shows phenotypic alignment with the core clinical manifestations of the TCM “phlegm-obstructed lung” syndrome, which suggests a hypothetical link between the TCM concept of “phlegm turbidity” and abnormal lipid-metabolite accumulation. In contrast, the LQD syndrome is marked by correlated downregulation of the lipid-transporter gene ABCA13. As an important member of the ABC transporter superfamily, ABCA13 is reported to be expressed in airway epithelial cells and responsible for transporting excess intracellular cholesterol and phospholipids to the extracellular space (42). Our serum-omics data cannot directly measure intracellular lipid deposition within airway epithelial cells; however, the observed reduction of ABCA13 transcript hypothetically implies compromised lipid transport capacity, which may reduce cell-membrane fluidity, disrupt airway epithelial-barrier integrity and weaken damage-repair capacity (43). This hypothetical pathological feature corresponds to the clinical manifestations of “lung-qi deficiency”, namely defective barrier defense and susceptibility to external pathogenic invasion, and this subtype may hypothetically correspond to the “epithelial barrier-deficient endotype” within asthmatic heterogeneity.

At the microecological level, no significant differences in α-diversity and overall community structure of oral microbiota were detected between the PZLP and LQD asthma syndromes, whereas PICRUSt2 predicted functional pathways showed obvious divergence between the two groups, providing correlational clues for understanding TCM syndrome heterogeneity. It should be emphasized that PICRUSt2 yields in-silico predicted microbial functional profiles instead of directly measured metabolic activities, and these predictions require further experimental validation. The oral-lung axis provides a plausible biological background for crosstalk between oral microbes and respiratory disease phenotypes, whereby oral microorganisms may produce neuroactive metabolites that potentially interfere with host neuro-immune responses (44, 45). In our dataset, the PZLP group was enriched in predicted serotonergic and dopaminergic synaptic-related pathways, and genera Bacillus and Cardiobacterium showed positive correlation with neuro-active metabolites and pro-inflammatory host genes. These correlational observations raise a testable hypothesis that a microbiota-neuro-immune axis might participate in PZLP-related pathological phenotypes, though causal evidence is absent from the present cross-sectional dataset. For the LQD syndrome, predicted methane and glycerol metabolic pathways were enriched, and the family Pasteurellaceae displayed significant negative correlation with host lipid-transporter gene ABCA13. This correlation cannot prove direct microbial regulation of ABCA13 expression; instead, it generates a hypothetical model in which microbial functional profiles may be linked to host lipid-transport-related molecular signatures. Correlation alone cannot distinguish whether microbial shifts drive host molecular changes, or host molecular status shapes oral microbial composition, or both are jointly modulated by other confounding clinical factors. Collectively, this multi-omics network connecting oral microbiota function, host metabolism and immune response offers correlational experimental evidence and testable mechanistic hypotheses for exploring microecological correlates of TCM asthma syndromes, rather than confirming definite host-microbe regulatory mechanisms (46). Further *in-vitro* co-culture experiments, germ-free animal models or longitudinal cohorts are needed to disentangle causal relationships between oral microbial features and TCM syndrome phenotypes.

The integrated research paradigm of “multi-omics combined with microecology” established in this study carries substantial theoretical innovations and clinical translational value. Theoretically, we systematically integrate the above three categories of omics findings to construct a multi-layer regulatory network underlying “same disease with different syndromes” in asthma. The combined detection panel constructed based on these biomarkers is expected to provide technical support for non-invasive, rapid and precise differentiation of TCM asthmatic syndromes in the future. More importantly, this study sheds light on individualized therapeutic strategies for asthma: for PZLP syndrome, therapeutic regimens combining SREBP-1c inhibitors with anti-inflammatory and expectorant agents could be explored as a hypothetical direction; for LQD syndrome, interventions focusing on ABCA13 agonists to improve lipid transport alongside airway epithelial barrier repair should be prioritized, providing experimental evidence for integrated Chinese and Western precision therapy for asthma.

Several inherent limitations of this study warrant acknowledgment. Initially, this single-center cross-sectional study with a relatively small sample size cannot determine causal links between molecular/microbial changes and the progression of TCM syndrome. Large-sample multicenter longitudinal cohort studies are required for external validation of our findings. Second, all mechanistic hypotheses proposed herein are derived from correlational multi-omics analysis of clinical patient samples. *In vitro* airway epithelial cell culture, organoid models, and syndrome-specific animal models are necessary to functionally validate the regulatory roles of ABCA13, PLD5, and key microbial functional pathways in syndrome pathogenesis. Third, this study exclusively profiled oral microbiota; although the oral-lung axis communication is widely recognized, the consistency of oral versus lower respiratory tract microbial signatures for TCM subtype discrimination requires further comparative verification. Admittedly, this exploratory study has a relatively small sample size, and model performance was evaluated by the built-in OOB validation rather than nested cross-validation. Future studies with expanded independent cohorts employing nested cross-validation are needed to confirm the diagnostic value of these oral microbial signatures.

In conclusion, integrated multi-omics profiling systematically characterizes correlational transcriptional, translational, metabolic, and oral-microbiome molecular signatures distinguishing healthy individuals and two core TCM subtypes of bronchial asthma. We propose a testable hypothesis that bidirectional lipid-metabolic imbalance constitutes a central correlational molecular feature differentiating PZLP and LQD syndromes, and demonstrate that oral-microbiota functional divergence hypothetically may mediate subtype-specific pathological progression via distinct regulatory pathways. These correlational findings deepen mechanistic comprehension of asthma pathological heterogeneity and pioneer novel research directions for objective TCM syndrome quantification and precision asthma diagnosis and intervention. Future research priorities include functional validation of the mechanistic hypotheses raised in this study and iterative development of multi-omics-biomarker-based diagnostic tools and individualized subtype-specific therapeutic regimens for asthma.

## Data Availability

The datasets presented in this study can be found in online repositories. The names of the repository/repositories and accession number(s) can be found below: PRJNA1484540 (NCBI SRA), MTBLS14938 (Metabolights) and PXD080635 (iPROX).
